# ST8Sia6 overexpression protects pancreatic β cells from spontaneous autoimmune diabetes in nonobese diabetic mice

**DOI:** 10.1172/JCI181207

**Published:** 2025-08-01

**Authors:** Justin Choe, Paul Belmonte, Sydney Crotts, Thanh Nguyen, David Friedman, Alexi Zastrow, Matthew Rajcula, Brady Hammer, Claire Wilhelm, Michael J. Shapiro, Aleksey Matveyenko, Virginia Smith Shapiro

**Affiliations:** 1 Department of Immunology and; 2 Department of Physiology and Biomedical Engineering, Mayo Clinic, Rochester, Minnesota, USA.

**Keywords:** Autoimmunity, Immunology, Autoimmune diseases, Beta cells, Diabetes

## Abstract

Type 1 diabetes is characterized by the autoimmune destruction of pancreatic β cells, resulting in permanent loss of glucose homeostasis. Islet transplantation is a promising potential cure that remains hindered by immune rejection. We previously showed that ST8Sia6 expression on tumors reduced immune surveillance and hypothesized that this sialyltransferase could protect β cells from autoimmune destruction. Here, we demonstrate that ectopic expression of ST8Sia6 in β cells of female nonobese diabetic mice (NOD βST) decreased the spontaneous incidence of diabetes by 90% and preserved β cell mass. NOD βST mice had comparable insulitis at 8 weeks of age that did not progress over time compared with littermate controls. β Cell–autoreactive B and T cells were present in NOD βST mice, indicating a peripheral rather than central mechanism of immune tolerance. The islets of NOD βST mice displayed a dampened type 1 immune response and reduced IL-12p35 expression in dendritic cells compared with those of littermate controls. The peripheral protection persisted even after removal of ST8Sia6 expression at 20 weeks of age, indicating that transient expression was sufficient for establishment of tolerance. These results demonstrate that ST8Sia6 protects β cells from immune-mediated attack and rejection, highlighting its therapeutic potential for autoimmune disorders.

## Abstract 2

Islet-specific autoimmune diabetes and inflammation is prevented in a mouse model when the enzyme ST8Sia6 is expressed in insulin-producing beta-cells

## Graphical abstract

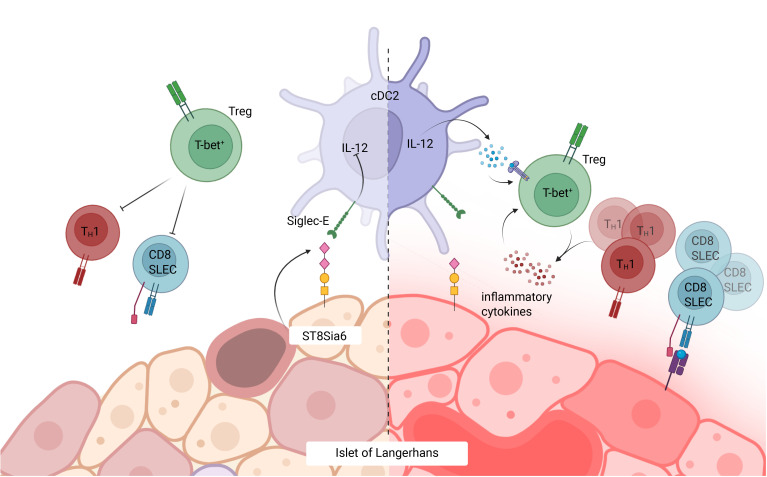

## Introduction

Type 1 diabetes (T1D) is an autoimmune disease caused by the selective destruction of the insulin-producing β cells within the islets of Langerhans in the endocrine pancreas (1). Insulin regulates blood glucose levels, and the irreversible loss of β cells results in a lifelong reliance on external insulin replacement. Poor disease management can lead to the acute medical emergency of diabetic ketoacidosis, while chronic imperfections in insulin dosing and delivery contribute to microvascular and macrovascular damage of the myocardium, kidneys, retinas, and peripheral extremities. The Diabetes Control and Complications Trial (2) demonstrated that intensive insulin therapy for tighter glycemic control mitigated some of the vascular complications of diabetes, but this benefit was offset by an increased risk of hypoglycemic episodes. The increasing prevalence of T1D (3%–4% globally over the past three decades) makes it a growing public health concern (1, 3).

Recent advances for managing T1D have largely focused on improved insulin delivery, including long-lasting insulin formulations (4) and open- or closed-loop pump systems (5, 6). These approaches improve patients’ time spent in the physiological blood glucose range (7) but still fail to perfectly normalize glucose homeostasis. In comparison, islet replacement therapy offers a potential cure, with freedom from regular glucose monitoring and insulin dosing. Transplantation of cadaveric islets into patients with diabetes resulted in more precise regulation of blood glucose (8) and demonstrated the most promise for long-term blood glucose normalization (9). Recently, progress has been made with the approval of donislecel for adults with T1D who struggle to manage disease via conventional means (10). However, to prevent allograft rejection of donor tissue and to suppress the established autoimmune response that precipitated disease, transplantation requires potent lifelong immunosuppression. Consequently, most patients experience adverse reactions, including but not limited to infection and malignancy (11). Compared with a systemic and generalized approach, targeted and localized immunomodulation of the immune response against β cells holds the potential to improve the success of curative islet transplantation and quality of life.

Sialyltransferases catalyze the addition of sialic acids as terminal modifications to cell surface glycans. This process occurs in the *trans*-Golgi and plays vital biological roles, including in cell-cell communication, recognition, differentiation, and fate determination (12). Sialic acids are also ligands for the Siglec (sialic acid–binding immunoglobulin-type lectin) family of receptors, which are primarily expressed on immune cells. Most of these receptors are inhibitory (13–15) and function by recruiting SHP-1 and SHP-2 tyrosine phosphatases to immunoreceptor tyrosine-based inhibitory motifs (ITIMs) or immunoreceptor tyrosine-based switch motifs (ITSMs) in their cytoplasmic tail upon ligand engagement (16). This signaling modality is exploited by both cancers (17) and pathogens (18, 19) to evade immune rejection. In addition, treatment of murine dendritic cells with sialylated OVA antigen in vitro generated antigen-specific regulatory T cells in a Siglec-dependent manner (20).

The sialyltransferase ST8Sia6 generates α2,8 disialic acids (21) that serve as the ligand for the murine inhibitory receptor Siglec-E (22) expressed by cells of the myeloid immune compartment (23). ST8Sia6 is expressed by many immune cells, including CD4^+^ T cells, B cells, macrophages, and dendritic cells (Immunological Genome Project [ImmGen]). Previously, we demonstrated that expression of ST8Sia6 enhanced tumor growth in the B16-F10 and MC38 cancer cell lines by modulating the immune response through Siglec-E and also accelerated tumorigenesis in a murine model of spontaneous colon cancer (24). As transgenic expression of ST8Sia6 protected tumor cells from an antitumor immune response, we considered whether this could be employed for protection of normal cells from an abnormal, pathologic immune response. Initially, we used the multiple-low-dose streptozotocin model of diabetes in C57BL/6 (B6) mice and found a delayed onset of hyperglycemia in mice with ST8Sia6 overexpressed in the β cells (22). However, this chemically induced model has limitations, including the uncertain role of autoreactive T cells in β cell loss (25), and likely fails to accurately reflect the spontaneous autoimmune response characteristic of human T1D.

The nonobese diabetic (NOD) mouse represents the most accurate model of the integrated multicellular immune response generated against β cell autoantigens in T1D patients. This model recapitulates human disease remarkably well, including genetic risk factors (26), autoantigens (27), and similar mechanisms of β cell rejection (28–30). Like T1D patients, NOD mice exhibit immune infiltration into the islet (insulitis) and the generation of autoantibodies prior to disease pathogenesis. Importantly, T cells are the effectors of β cell destruction in the NOD mouse. The vital role of T cells in T1D is highlighted by clinical trials that demonstrated a delay in the onset of symptomatic diabetes in high-risk (31) and newly diagnosed (32) individuals after treatment with teplizumab, an anti-CD3ε monoclonal antibody. For these reasons, the NOD mouse remains the gold standard for modeling T1D.

Drawing inspiration from cancer’s exploitation of immunomodulatory mechanisms, we sought to demonstrate that ST8Sia6 could abrogate spontaneous autoimmunity for therapeutic benefit. Here, we engineered a transgenic model to conditionally express ST8Sia6 in the β cells of NOD mice (NOD βST mice, for β cells expressing ST8Sia6). ST8Sia6 is not normally expressed in β cells (from Tabula Muris, Tabula Sapiens, or UCSC Cell Browser) (33–35). There was a 90% reduction in the development of autoimmune diabetes and preservation of β cell mass within the pancreas in NOD βST mice. There was local protection of islets from immune destruction, despite the presence of autoreactive T and B cells against β cells and ongoing Sjögren’s syndrome characterized by unimpaired immune infiltration into the salivary glands in NOD βST mice. The peripheral protection persisted even after removal of ST8Sia6 expression at 20 weeks of age, indicating that transient expression was sufficient for establishment of tolerance. We found reductions in adaptive mediators of the type 1 response and decreased IL-12p35 expression by type 2 conventional dendritic cells in the islets of NOD βST mice. Our results point toward the potential of this approach to mitigate and possibly cure autoimmune diabetes through Siglec–sialic acid interactions.

## Results

### Expression of ST8Sia6 in the islets of female NOD mice protects from spontaneous disease.

**Figure 1:**
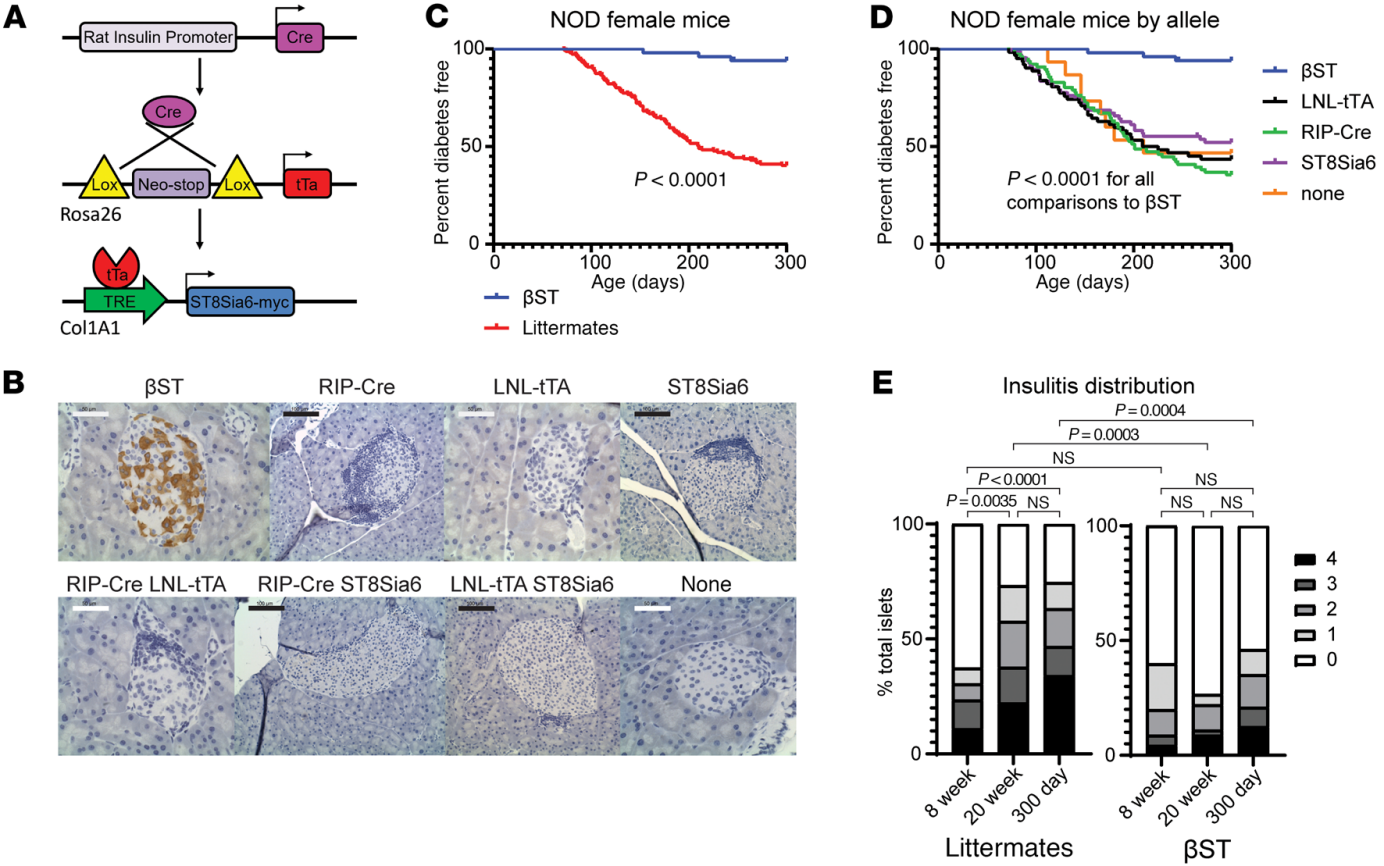
Expression of ST8Sia6 in the islets of NOD female mice protects from spontaneous disease. (**A**) Schematic representation of the genetic model. (**B**) Representative IHC imaging for the myc-tagged ST8Sia6 in pancreas sections from each genetic combination of alleles in female mice. Only βST mice expressing all 3 alleles stain positive for myc-tagged ST8Sia6 in the islets. Scale bars: 100 μm (black) or 50 μm (white). (**C**) Kaplan-Meier curve for diabetes-free incidence in female NOD βST and NOD littermate mice. *n* = 50 (NOD βST) or 151 (littermates). Statistical significance was determined by log-rank Mantel-Cox test. (**D**) Subanalysis by allelic expression of the littermate population from **C** for diabetes-free incidence. *n* = 50 (NOD βST), 62 (LNL-tTA), 76 (RIP-Cre), 67 (ST8Sia6), or 15 (no alleles); the sum of littermate subgroups exceeds 151 because some littermates carried 2 alleles and were thus counted in more than one group. Statistical significance was determined by log-rank Mantel-Cox test comparing each littermate group against NOD βST. (**E**) Assessment of the extent of immune infiltration into the islets of nondiabetic female NOD βST and littermate mice at 8 weeks, 20 weeks, and 300 days of age. Insulitis was scored along a 4-point scale (0, no insulitis; 1, 0%–25% infiltration; 2, 26%–50% infiltration; 3, 51%–75% infiltration; 4, 76%–100% infiltration) from H&E-stained sections. Analysis was performed on 45 islets from 4 βST and 72 islets from 7 littermate mice at 8 weeks; 50 islets from 6 βST and 59 islets from 12 littermate mice at 20 weeks; and 119 islets from 16 βST and 76 islets from 17 littermate mice at 300 days. Distribution of scores is plotted, and statistical analysis was performed using a χ^2^ test on islet scores for indicated comparisons.

Previously, we generated a mouse model in the B6 background to drive the expression of ST8Sia6 in β cells in a Cre-dependent and doxycycline-regulatable manner (βST mice) (22). To determine whether expression of ST8Sia6 could protect β cells in a genetic model of spontaneous T1D, we backcrossed our βST mouse from the B6 background to the NOD model for at least 10 generations, establishing the NOD βST mouse. In the 3-allele system, described in Figure 1A, the insulin promoter drives Cre recombinase (RIP-Cre) to delete a neomycin cassette, allowing for the expression of the tet-transactivator (LNL-tTA), which initiates expression of a myc-tagged ST8Sia6 transgene. β Cells expressed ST8Sia6 only when all 3 alleles were present (Figure 1B), while littermates with 2 or fewer alleles did not express ST8Sia6 (referred to as NOD littermates). We monitored NOD βST mice and littermate controls by twice-weekly nonfasting tail vein blood glucose readings and defined diabetes as 3 consecutive readings of at least 250 mg/dL. Female NOD βST mice were protected from spontaneous diabetes compared with NOD littermate controls (Figure 1C), with 6% (3/50) diabetes incidence in NOD βST mice compared with nearly 60% (90/151) in littermate controls over the study course of 300 days. Importantly, a subanalysis of female littermate outcomes demonstrated that no single allele affected diabetes onset (Figure 1D). This finding showed that the protection from disease we observed required all 3 alleles resulting in ST8Sia6 expression in β cells to provide a 90% protection from the development of diabetes in female NOD mice.

### Expression of ST8Sia6 in the islets of male NOD mice also protects from spontaneous disease.

Male NOD βST mice were also protected from spontaneous diabetes, with 18% (10/55) disease incidence compared with 47% (58/124) in male NOD littermates (Supplemental Figure 1A; supplemental material available online with this article; https://doi.org/10.1172/JCI181207DS1). This difference in male diabetes incidence was not as pronounced as in females, which may be partially attributed to previously described sex bias of disease toward females in the NOD model (36, 37). However, subanalysis of male NOD littermate outcomes showed that those expressing the ST8Sia6 allele (with or without RIP-Cre or LNL-tTA) fared as well as their βST counterparts (Supplemental Figure 1B) with only 26% (17/66) disease incidence. Male NOD littermates not expressing the ST8Sia6 allele demonstrated over 70% (41/58) diabetes incidence. IHC staining for the myc tag revealed unexpected ST8Sia6 expression in the islets of male NOD ST8Sia6 allele–positive mice (Supplemental Figure 1C) that did not occur in NOD female mice (Figure 1B); this was not observed in islets of male B6 mice carrying the same ST8Sia6 allele under the control of a gut epithelial cell–specific Cre (Supplemental Figure 1D). Thus, the expression of ST8Sia6 without concurrent expression of RIP-Cre and LNL-tTA was specific to the male NOD mouse. While the mechanism responsible remains unclear, these findings indicate that β cell expression of ST8Sia6 protected from T1D in both female and male NOD mice. The unexpected expression of ST8Sia6 in male NOD mice along with documented sex bias in disease (36, 37) led us to focus only on female mice for the remainder of the present study.

### Progression of the disease process differs between βST and littermate mice.

One potential mechanism for protection involves the inability of autoreactive immune cells to infiltrate the islet. NOD mice experience a well-documented progression of insulitis, initiating at approximately 4 weeks of age with gradual amplification of the immune response until the extent of β cell destruction results in loss of glucose homeostasis (38). To assess potential differences in this time course of immune infiltration into the islets, pancreata from euglycemic NOD βST and littermate mice were harvested for histologic assessment at 8 weeks, 20 weeks, and the study endpoint of 300 days. A 4-point scale was used to assess the severity of peri-islet insulitis; islets were scored either 0 (no insulitis), 1 (0%–25% immune infiltration), 2 (26%–50% infiltration), 3 (51%–75% infiltration), or 4 (up to 100% infiltration). Examples of insulitis scoring are in Supplemental Figure 2A. Both NOD βST and littermate mice at 8 weeks of age demonstrated mild insulitis, with most of the islets scored 0 at this age (Figure 1E). As expected, insulitis progressed over time in the NOD littermate controls. By contrast, NOD βST islets from 20 weeks and 300 days of age continued to show mild insulitis that did not differ significantly from the insulitis observed at 8 weeks of age (Figure 1E). While there was a slight increase in the number of islets that scored 4 in βST mice at 300 days, this represented only 12.6% of all islets; in contrast, most islets in this group remained protected, with over 50% still showing no insulitis and scored 0. Thus, while female NOD βST mice demonstrated initial insulitis, there was an absence of progression over time.

### β Cells in the islets of NOD βST mice are preserved.

**Figure 2:**
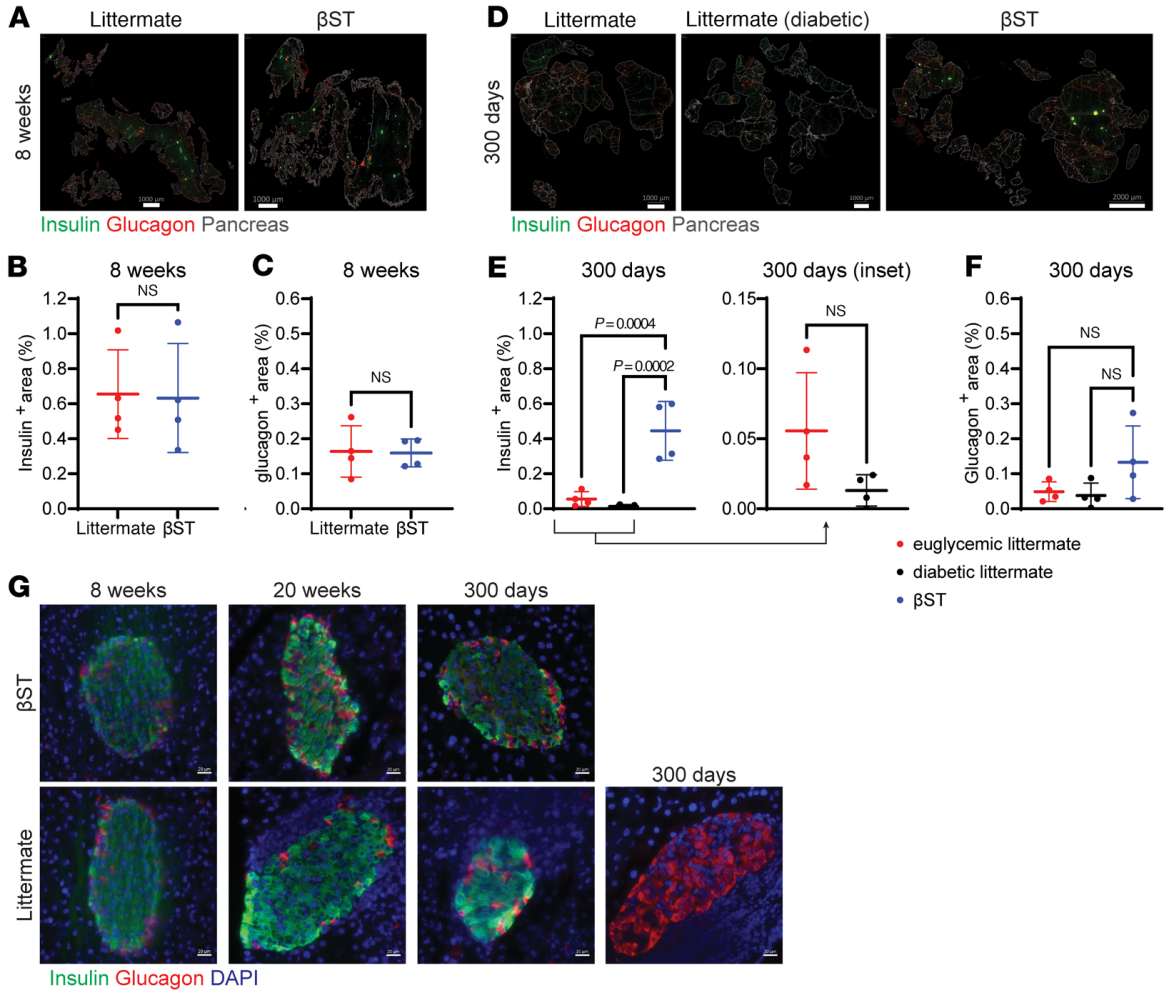
Disease modulation is secondary to preservation of β cells in the islets. (**A**) Representative immunofluorescence images of whole pancreas sections from 8-week-old nondiabetic NOD βST and NOD littermate mice. (**B** and **C**) Quantification of insulin^+^ (green; FITC) area (**B**) and glucagon^+^ (red; Cy3) area (**C**) in the pancreata of 8-week-old nondiabetic NOD βST and littermate mice (*n* = 4 per group). Error bars represent standard deviation (SD) from the mean. Mann-Whitney *U* test was used for comparisons. (**D**) Representative immunofluorescence images of whole pancreas sections from 300-day-old nondiabetic NOD βST, nondiabetic NOD littermate, and diabetic NOD littermate mice. (**E** and **F**) Quantification of insulin^+^ (**E**) and glucagon^+^ (**F**) area in the pancreata of 300-day-old mice (*n* = 4 per group). One-way ANOVA was performed for statistical analysis between all 3 groups, with Mann-Whitney *U* test for inset comparison. Scale bars: 1,000 μm, except rightmost panel of **D**: 2,000 μm. (**G**) Immunofluorescence images of histologic sections of pancreas from euglycemic NOD βST and NOD littermate mice at the indicated ages with insulin in green (FITC), glucagon in red (Cy3), and DAPI staining for nuclei. Four pancreata from each group at each indicated age were stained and imaged, and representative islets are presented. One islet from a NOD littermate at 300 days of age demonstrates loss of insulin but preservation of glucagon. Scale bars: 20 μm.

We wanted to investigate whether β cell mass was preserved in NOD βST mice. To determine a baseline measure of β cells, sections of pancreata from 8-week-old nondiabetic NOD βST and littermate mice were analyzed for insulin and glucagon to quantify β cell and α cell area, respectively (Figure 2, A–C). At this early time point when insulitis was not yet severe in either group (Figure 1E), NOD βST and littermate mice exhibited a comparable percentage of the pancreas cross-sectional area that was insulin and glucagon positive.

A similar analysis was conducted in pancreas sections from nondiabetic NOD βST, nondiabetic NOD littermate, and diabetic NOD littermate mice at the study endpoint of 300 days (Figure 2, D–F). As expected, diabetic NOD littermates demonstrated a near-total loss of β cell mass. There was also a substantial decrease in the insulin-positive area even in euglycemic NOD littermates at this age. In comparison, the insulin-positive area within the pancreas was largely preserved in 300-day-old NOD βST mice as compared with the analysis from the 8-week time point (Mann-Whitney *U* test, *P* = 0.38). The glucagon-positive area in the pancreas did not differ significantly between these groups at the 300-day time point.

The islets in NOD βST mice were morphologically similar to the islets in NOD littermate mice (Figure 2G) with similar composition and distribution of β cells and α cells (39). These islets were selected from euglycemic mice for morphologic assessment, and some islets from littermates demonstrated ongoing disease process indicated by insulitis (DAPI-dense peri-islet immune infiltration) or, in an extreme case, complete loss of insulin-positive β cells.

Eight-week-old NOD βST mice responded similarly to littermates when challenged with a bolus of glucose or insulin, with similar initial (0 minutes) and final (120 minutes) blood glucose levels and similar kinetics of response in both an intraperitoneal glucose tolerance test (IPGTT) and an intraperitoneal insulin tolerance test (IPITT) (Supplemental Figure 2, C–E). Importantly, these kinetics were not influenced by the weights of the mice, which were similar between NOD βST and littermate mice (Supplemental Figure 2B). Additionally, expression of RIP-Cre in littermates did not alter the IPGTT response (Supplemental Figure 2D). No differences in serum insulin or C-peptide were observed between euglycemic NOD βST mice and littermate controls between 8 and 10 weeks of age (Supplemental Figure 2, F and G).

Colocalization of the myc-tagged ST8Sia6 transgene and insulin was observed in NOD βST mice (Supplemental Figure 3). This colocalization did not change over time (Supplemental Figure 3B) or with increasing insulitis (Supplemental Figure 3C). As expected from H&E profiling (Figure 1E), NOD βST islets did not show statistically significant insulitis. Thus, despite similar β cell mass in the pancreas early in life, only NOD βST mice showed preservation over time in comparison with littermate controls.

### Protection from autoimmunity in βST mice is localized to the islet.

**Figure 3:**
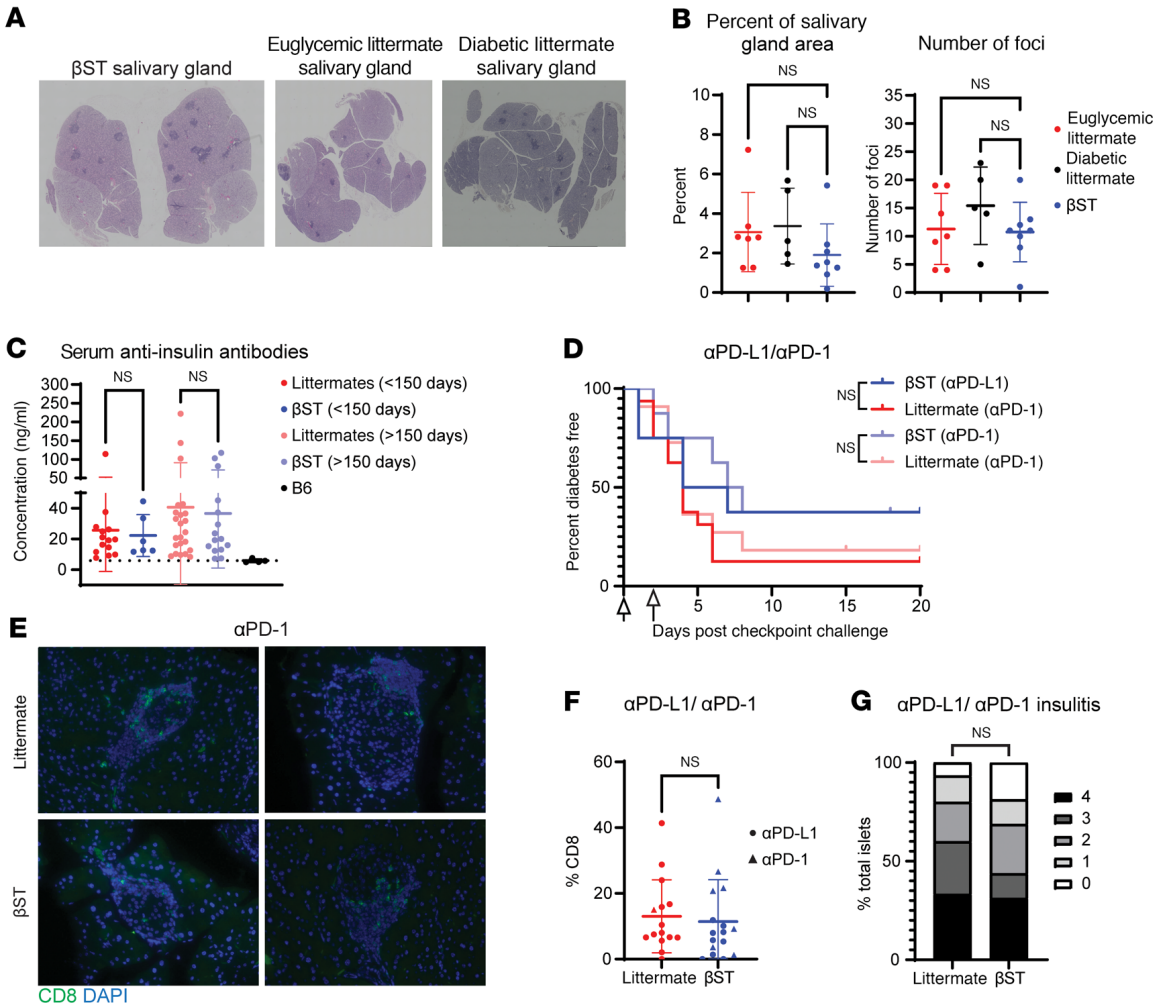
Protection from autoimmunity in βST mice is localized to the islet and peripheral in nature. (**A**) Representative H&E staining of salivary glands from female 300-day-old nondiabetic NOD βST mice or nondiabetic or diabetic littermate controls. Images are stitched composites (original magnification, ×5). (**B**) Number of foci of immune infiltrates and percentage area of immune foci in salivary glands of 7 nondiabetic NOD littermate, 5 diabetic NOD littermate, or 8 euglycemic NOD βST mice. Error bars represent SD, and 1-way ANOVA was used for comparisons. (**C**) ELISA quantification of serum anti-insulin antibodies from nondiabetic female NOD βST and littermate mice. Mice were stratified by age into the first half of the disease kinetics study (6 NOD βST and 14 littermates) or the latter half (15 NOD βST or 23 littermates). Serum from B6 mice (*n* = 4) was analyzed to determine threshold of detection. Error bars represent SD, and Mann-Whitney *U* test was used for indicated comparisons. (**D**) Diabetes-free incidence in 16-week-old female euglycemic mice challenged intraperitoneally with anti–PD-L1 (8 NOD βST and 16 littermates) or anti–PD-1 (8 NOD βST and 11 littermates). Log-rank Mantel-Cox test was performed to analyze statistical differences between indicated groups. (**E**) Representative immunofluorescence images of CD8 in pancreas from NOD βST and littermate mice after challenge with anti–PD-1 from **D**. Original magnification, ×2 (islets ). (**F**) Quantification of CD8^+^ T cell infiltration in islets from pancreas sections from **D**. *n* = 15 islets from 3 NOD littermate pancreas sections (14 islets from 1 mouse challenged with anti–PD-L1, 1 islet from 1 mouse challenged with anti–PD-1) or 16 islets from 4 NOD βST pancreas sections (6 islets from 2 mice challenged with anti–PD-L1, 9 islets from 2 mice challenged with anti–PD-1). Error bars represent SD. Mann-Whitney *U* test was used for indicated comparison. (**G**) Insulitis distribution in pancreas sections from **F** scored and analyzed as in Figure 1E.

In addition to modeling T1D, the NOD mouse is a well-described model of Sjögren’s syndrome (40), another autoimmune disorder characterized by immune infiltration into glandular tissues such as the salivary and lacrimal glands. To determine whether β cell–specific ST8Sia6 expression led to systemic changes in the autoimmune landscape, submaxillary salivary glands from euglycemic 300-day-old female NOD βST as well as euglycemic and diabetic littermate mice were analyzed for immune infiltration (Figure 3A). Neither the number of immune foci nor the relative percentage area of the gland occupied by immune infiltration differed between euglycemic or diabetic NOD littermates and NOD βST mice (Figure 3B). In addition, there was no increase in immune infiltration into the salivary glands in diabetic as compared with euglycemic littermate control mice. Therefore, transgenic expression of ST8Sia6 provided protection specifically to β cells but did not alter the immune infiltration into the salivary glands in NOD mice.

Prior to the development of overt T1D, patients exhibit detectable autoantibodies against islet antigens, including insulin (41). This is also observed in NOD mice, with detectable serum autoantibodies against many similar β cell autoantigens (27), though their role in disease pathogenesis is not well understood. The concentration of serum autoantibodies against insulin was quantified in either the first half (0–150 days) or the latter half (151–300 days) of the study in nondiabetic NOD βST and littermate mice, with serum from euglycemic B6 mice used to establish the lower threshold of detection. In either time frame, similar levels of anti-insulin antibodies were observed between NOD βST and littermate controls (Figure 3C). These findings suggest that ST8Sia6 expression in β cells did not preclude the peripheral generation of autoantibodies in germinal center reactions of pancreatic lymph nodes, which are important clinical biomarkers that identify risk of progression to fulminant disease (42).

Another consideration was that the ST8Sia6 transgene altered thymic selection, leading to a failure to generate islet-autoreactive T cells that could mediate β cell destruction. The autoimmune regulatory element (AIRE) has been demonstrated to regulate access of peripheral tissue promoters, including the insulin promoter (reviewed in ref. 43). Anti–PD-L1 or anti–PD-1 challenge was previously demonstrated to break tolerance in euglycemic NOD mice, resulting in rapid development of diabetes (44, 45). To test the possibility that the protection observed in our NOD βST mice was secondary to enhanced negative selection of immature autoreactive thymocytes, an immune checkpoint inhibition challenge was conducted. Euglycemic NOD βST and littermate mice at 14–16 weeks of age were given 1 or 2 doses of anti–PD-L1 or anti–PD-1 and monitored daily for hyperglycemia. Both groups rapidly developed diabetes (Figure 3D), demonstrating the presence of autoreactive T cells in the periphery of both NOD βST and NOD littermate mice. Pancreatic sections were examined from anti–PD-L1– or anti–PD-1–challenged NOD βST mice and littermate controls, and similar infiltration of CD8^+^ T cells was observed in islets from these mice (Figure 3E, quantified in Figure 3F). Additionally, islets from NOD βST mice had a similar severity of insulitis after immune checkpoint inhibition compared with littermate controls (Figure 3G). The presence of anti-insulin antibodies and autoreactive T cells in NOD βST mice indicated a peripheral mechanism of protection from disease rather than central tolerance.

### NOD βST mice have a reduction in the type 1 immune response in the islet.

T1D, in both the NOD mouse and humans, is characterized by a potent type 1 immune response (27); Th1 CD4^+^ T cells orchestrate inflammation while cytotoxic effector CD8^+^ T cells mediate β cell death. To analyze the type 1 response, the spleen, draining pancreatic lymph nodes (PLNs), and islets from nondiabetic NOD βST and nondiabetic littermate mice were harvested and analyzed via flow cytometry. These tissues were profiled from mice at the intermediate age of 14 weeks, which is the age when NOD littermates began developing diabetes (Figure 1C). The gating scheme to analyze Th1 cells (T-bet^+^CD4^+^), Th17 cells (RORγt^+^CD4^+^), short-lived effector CD8^+^ T cells (SLECs; T-bet^+^CD8^+^), and regulatory T cells (Tregs; FoxP3^+^CD4^+^) from islets is shown in Supplemental Figure 4A (gating scheme for PLNs in Supplemental Figure 4B); Tregs were further analyzed according to T-bet expression, as T-bet^+^ Tregs have been previously shown to strongly suppress type 1 inflammation and diabetes in the NOD model (46).

**Figure 4:**
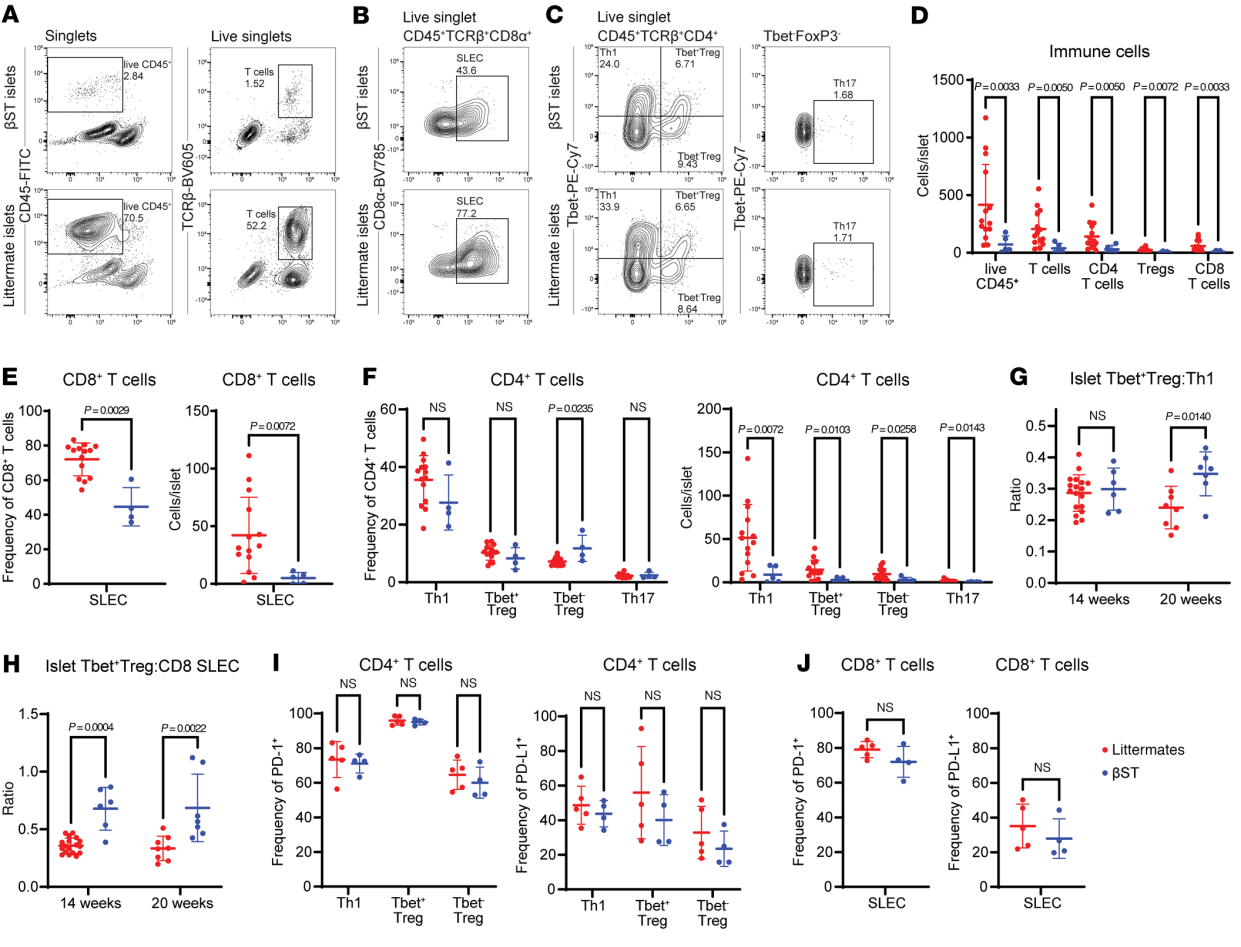
βST mice have a reduction in the type 1 immune response in the islet. (**A**–**C**) Representative flow cytometry plots of immune cells (**A**), short-lived effector CD8^+^ T cells (SLECs) (**B**), and CD4^+^ T cell subsets (**C**) in islets from euglycemic NOD βST or littermate mice at 14 weeks of age. Cells were previously gated by size and singlets. Within the live singlet CD45^+^TCRβ^+^CD4^+^ cell population, Th1 cells (FoxP3^–^T-bet^+^), Th17 cells (FoxP3^–^RORγt^+^), and Tregs (parsed by T-bet^+^ and T-bet^–^) were analyzed. SLECs were defined as T-bet^+^ cells within the live singlet CD45^+^TCRβ^+^CD8^+^ cell population. (**D**) Quantification of normalized event count of cells depicted in **A**. The number of each cell type was normalized to the number of cells per islet per mouse from 5 NOD βST or 14 littermates. (**E**) Quantification of frequency and normalized cell count of SLECs per islet isolated per mouse. One NOD βST mouse was censored because of lack of insulitis. (**F**) Quantification of frequency and normalized cell count of CD4^+^ T cell subsets per islet from **C** isolated per mouse; one NOD βST mouse was censored because of lack of insulitis. (**G**) Ratio of T-bet^+^ Tregs (T-bet^+^FoxP3^+^CD4^+^) to Th1 T cells (T-bet^+^FoxP3^–^CD4^+^) from the islets of euglycemic NOD βST or NOD littermate mice at 14 weeks as above and 20 weeks of age (7 NOD βST or 14 littermate mice). (**H**) Ratio of T-bet^+^ Tregs (T-bet^+^FoxP3^+^CD4^+^) to SLECs (T-bet^+^CD8^+^) from the islets of NOD βST or NOD littermate mice as in **G**. (**I** and **J**) Quantification of PD-1 and PD-L1 expression in CD4^+^ T cell subsets (**I**) and SLECs (**J**) from 4 NOD βST or 5 littermates. Error bars represent SD. Mann-Whitney *U* tests were used to examine statistical differences between groups.

A reduction in overall immune infiltration (CD45^+^ cells) was found in the islets of NOD βST mice compared with littermate controls (Figure 4, A and D), consistent with the histologic analysis of insulitis (Figure 1E). These data were normalized to the number of cells per islet (Figure 4D), with a similar number of islets isolated from both NOD βST mice and littermate controls for this analysis (Supplemental Figure 4C). Similar reductions were also observed in T cells (live single CD45^+^TCRβ^+^), including CD4^+^ T cells, CD8^+^ T cells, and Tregs, in the islets of NOD βST mice compared with littermate controls (Figure 4, A and D). No differences in these populations were observed in the PLNs (Supplemental Figure 5, A and D) or spleens (Supplemental Figure 6, A and D) of NOD βST and littermate mice.

NOD βST mice demonstrated significant reductions in both the frequency and absolute number of CD8^+^ SLECs per islet (Figure 4, B and E). NOD βST mice also demonstrated a slight reduction (albeit not statistically significant) in the frequency of Th1 cells and an increased frequency of T-bet^–^ Tregs (Figure 4, C and F) in the islets compared with littermates. When the absolute number of these cells was analyzed per islet, all profiled CD4^+^ populations were reduced in the NOD βST mice compared with NOD littermates, with a striking reduction in the number of Th1 cells in the NOD βST mice (Figure 4F). These reductions in the type 1 response were specific to the islets of NOD βST mice, as no differences were noted in either the frequency or number of Th1, Th17, T-bet^+^, or T-bet^–^ Tregs or CD8^+^ SLECs in the PLNs (Supplemental Figure 5, B, C, E, and F) or spleens (Supplemental Figure 6, B, C, E, and F) between NOD βST and littermate mice. We also examined PD-1 and PD-L1 expression in CD4^+^ Th1 cells, T-bet^+^ and T-bet^–^ Tregs, and CD8^+^ SLECs from the islets, PLNs, and spleens of NOD βST and NOD littermate mice. No differences were observed in any of these populations in any of these tissues (Figure 4, I and J, Supplemental Figure 5, G and H, and Supplemental Figure 6, G and H; representative islet histograms in Supplemental Figure 4D).

Previous work demonstrated that T-bet^+^CXCR3^+^ Tregs were particularly effective at suppressing inflammation and played an important role in the inhibition of diabetes development in NOD mice (46). As disease progresses, the ratio of Tregs to T effectors decreases. In the islets of our NOD βST mice, the ratio of T-bet^+^FoxP3^+^ Tregs to T-bet^+^ Th1 CD4^+^ T cells or T-bet^+^ CD8^+^ SLECs was significantly higher in comparison with littermate controls at 20 weeks of age. At 14 weeks of age, only the ratio of T-bet^+^FoxP3^+^ Tregs to T-bet^+^ CD8^+^ SLECs was significantly higher in the NOD βST islets (Figure 4, G and H). No differences in these ratios were observed in the draining PLNs at either 14 or 20 weeks (data not shown). Thus, the expression of ST8Sia6 in islets promoted a tolerant environment, at least in part through sustained Treg to T effector ratios in the islets.

### βST mice have reduced IL-12p35 expression in type 2 conventional dendritic cells.

**Figure 5:**
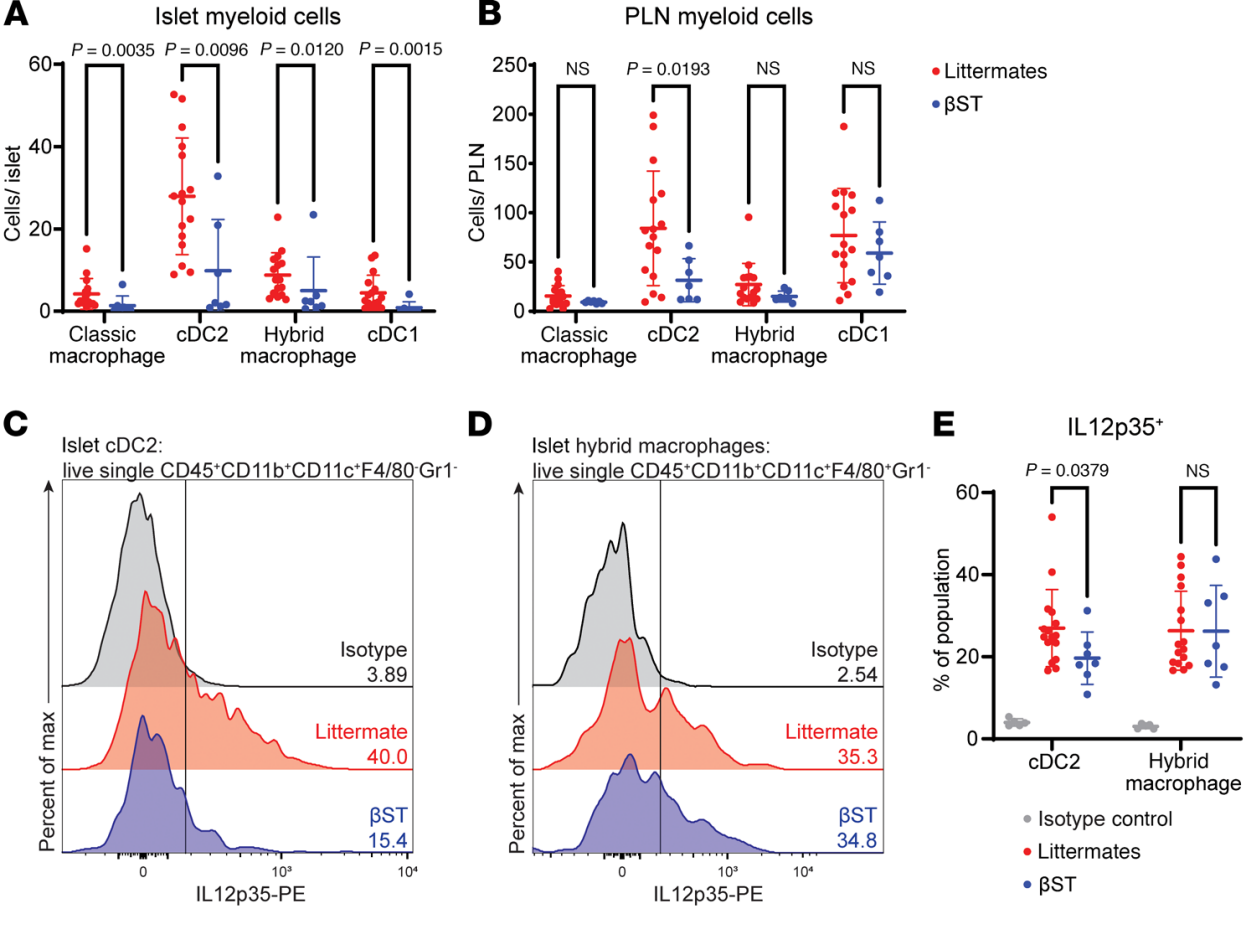
βST mice have reduced IL-12p35 expression in islet myeloid cells. (**A** and **B**) Normalized cell count of classical macrophages (CD11b^+^CD11c^–^F4/80^+^Gr1^–^), hybrid macrophages (CD11b^+^CD11c^+^F4/80^+^Gr1^–^), cDC1s (CD11b^–^CD11c^+^F4/80^–^Gr1^–^), and cDC2s (CD11b^+^CD11c^+^F4/80^–^Gr1^–^) per islet (**A**) or per pancreatic lymph node (**B**) from euglycemic 14-week-old NOD βST or littermate mice. (**C**) Representative flow cytometry histogram of IL-12p35 expression in cDC2s from islets of euglycemic 14-week-old NOD βST and NOD littermate mice, compared with isotype control. (**D**) Representative flow cytometry histogram of IL-12p35 expression in hybrid macrophages from islets of euglycemic NOD βST and littermate mice, compared with isotype control. Vertical line denotes the positive gate. (**E**) Quantification of IL-12p35 expression shown in **C** and **D** across 7 NOD βST mice, 16 NOD littermate mice, and 5 isotype controls. Error bars represent SD. Mann-Whitney *U* tests were used to examine statistical differences between groups.

To elucidate the underlying mechanism responsible for driving the local reduction in the type 1 response, myeloid populations in the islets of nondiabetic NOD littermate mice were profiled. Expression of Siglec-E, a murine-specific Siglec receptor previously demonstrated to be able to engage ST8Sia6-generated ligands (22), is restricted to myeloid cells. Macrophage and dendritic cell subsets in the spleen, PLNs, and islets from euglycemic 14-week-old NOD βST and littermate mice were analyzed via flow cytometry for classical macrophages (CD11b^+^CD11c^–^F4/80^+^Gr1^–^), hybrid macrophages (CD11b^+^CD11c^+^F4/80^+^Gr1^–^), type 2 conventional dendritic cells (cDC2s: CD11b^+^CD11c^+^F4/80^–^Gr1^–^), and type 1 conventional dendritic cells (cDC1s: CD11b^–^CD11c^+^CD8α^+^) (gating scheme in the islets and PLNs is shown in Supplemental Figure 7, A and B). As expected from previous observations (Figure 1E and Figure 4A), the islets of NOD βST mice demonstrated significant reductions in the number of each of these myeloid populations (Figure 5A) compared with littermate controls, despite similar islet yield for analysis (Supplemental Figure 7C). Interestingly, cDC2s (but no other myeloid population) were decreased in the draining PLNs of NOD βST mice as compared with littermate controls (Figure 5B). This was not surprising, as cDC2s are migratory (47), and a decrease in the draining lymph nodes of NOD littermate mice would be expected given the observed decrease in this population in the islets.

Of the myeloid populations profiled, only cDC2s (Supplemental Figure 8A) and hybrid macrophages (Supplemental Figure 8B) isolated from both the islets and PLNs were found to express Siglec-E. cDC1s do not express Siglec-E (ImmGen), and there were too few classic macrophages in the islet to analyze Siglec-E expression in that population. IL-12 is a potent type 1 cytokine responsible for disease pathogenesis in the NOD mouse, with the α subunit (also known as IL-12p35) playing a vital role. In the NOD mouse, a knockout of the α subunit (48) but not the β subunit (49) resulted in protection from diabetes. In addition, NOD IL-12p35–heterozygous mice had an intermediate level of protection from diabetes, demonstrating the critical importance of IL-12p35 levels in disease progression. This translates to humans: serum IL-12 was positively correlated with the development of T1D in neonates and children, and negatively correlated with circulating Treg frequency in patients with recent-onset diabetes (50, 51). Thus, we measured IL-12p35 expression in myeloid subsets, given the key role this cytokine plays in promoting type 1 immune responses.

In cDC2s, which are the major myeloid population in the islets of NOD mice, significantly lower levels of IL-12p35 in NOD βST mice compared with NOD littermate mice were observed (Figure 5, C and E). There was not a significant difference in IL-12p35 expression in hybrid macrophages from these same islets (Figure 5, D and E). No reductions in IL-12p35 expression were noted in cDC2s or hybrid macrophages from the PLNs of NOD βST mice compared with littermates (Supplemental Figure 8, C–E). Altogether, these findings demonstrated a localized alteration to inflammatory cytokine production by migratory cDC2s at the site of ST8Sia6 expression in the islets, possibly through engagement of the immunomodulatory receptor Siglec-E. This reduction in IL-12p35 by cDC2s could contribute to the mitigation of the type 1 immune response that characterizes β cell rejection in T1D.

### Exogenous IL-12 normalizes frequency and ratio of immune populations in NOD βST mice.

**Figure 6:**
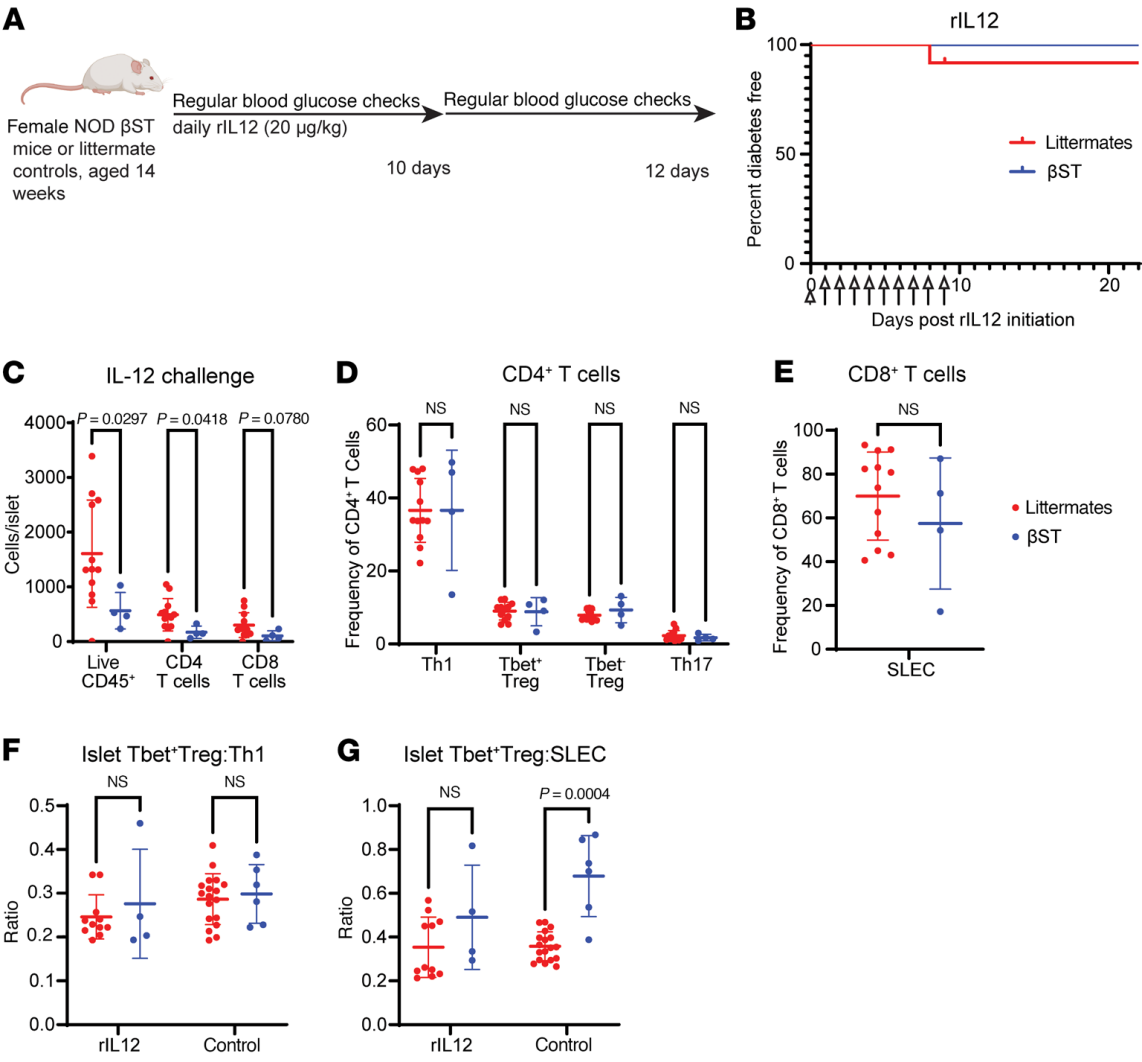
NOD βST mice challenged with recombinant IL-12 demonstrate increased islet inflammation. (**A**) Schematic of IL-12 challenge. Euglycemic 14-week-old female NOD βST and littermate mice were injected intraperitoneally with 20 μg/kg of IL-12 daily for 10 days (or until hyperglycemic). (**B**) Diabetes-free incidence in these mice over 3 weeks. *n* = 4 (NOD βST) or 12 (NOD littermates). (**C**) Quantification of normalized counts of all immune cells, CD4^+^ T cells, and CD8^+^ T cells in the islets of mice challenged with IL-12 at the study endpoint of 3 weeks. (**D** and **E**) Quantification of frequency of CD4^+^ T cell subsets (**D**) and CD8^+^ SLECs (**E**) in the islets of these mice. (**F**) Ratio of T-bet^+^ Tregs (T-bet^+^FoxP3^+^CD4^+^) to Th1 T cells (T-bet^+^FoxP3^–^CD4^+^) from the islets of these mice challenged with IL-12, compared with the same ratios from euglycemic 14-week-old mice (control from Figure 4G). (**G**) Ratio of T-bet^+^ Tregs (T-bet^+^FoxP3^+^CD4^+^) to SLECs (T-bet^+^CD8^+^) from the islets of these mice challenged with IL-12, compared with the same ratios from euglycemic 14-week-old mice (control from Figure 4H). Same gating scheme as Figure 4. Error bars represent SD. Mann-Whitney *U* test was used to examine statistical differences between groups.

Administration of exogenous IL-12 has previously been shown to induce hyperglycemia in NOD mice (52). We injected 14-week-old nondiabetic NOD βST mice and littermate controls with IL-12 daily for 10 days (schematically shown in Figure 6A). Although induction of overt hyperglycemia was not observed (Figure 6B), IL-12 administration did alter the frequency and ratio of immune populations in the NOD βST mice to resemble those in their littermate controls. The total numbers of CD45^+^ cells, CD4^+^ T cells, and CD8^+^ T cells per islet remained reduced in NOD βST mice (Figure 6C), but the frequencies of CD4^+^ Th1 cells, T-bet^+^ Tregs, T-bet^–^ Tregs, Th17 cells, and CD8^+^ SLECs were similar after IL-12 administration (Figure 6, D and E). Interestingly, the ratios of T-bet^+^FoxP3^+^ Tregs to T-bet^+^ Th1 CD4^+^ T cells or CD8^+^ SLECs were similar between IL-12–treated NOD βST mice and littermate controls (Figure 6, F and G; controls from Figure 4, G and H, for comparison); this is in contrast to the previously observed increased ratio of T-bet^+^FoxP3^+^ Tregs to CD8^+^ SLECs in nontreated NOD βST mice compared with littermate controls (Figure 4H). Thus, the decreased immune activation observed in the islets of NOD βST mice can be overcome by administration of exogenous IL-12, supporting our original conclusions that decreased IL-12p35 expression may contribute to the dampened type 1 immune response in βST mice.

### NOD βST mice establish tolerance after 20 weeks.

**Figure 7:**
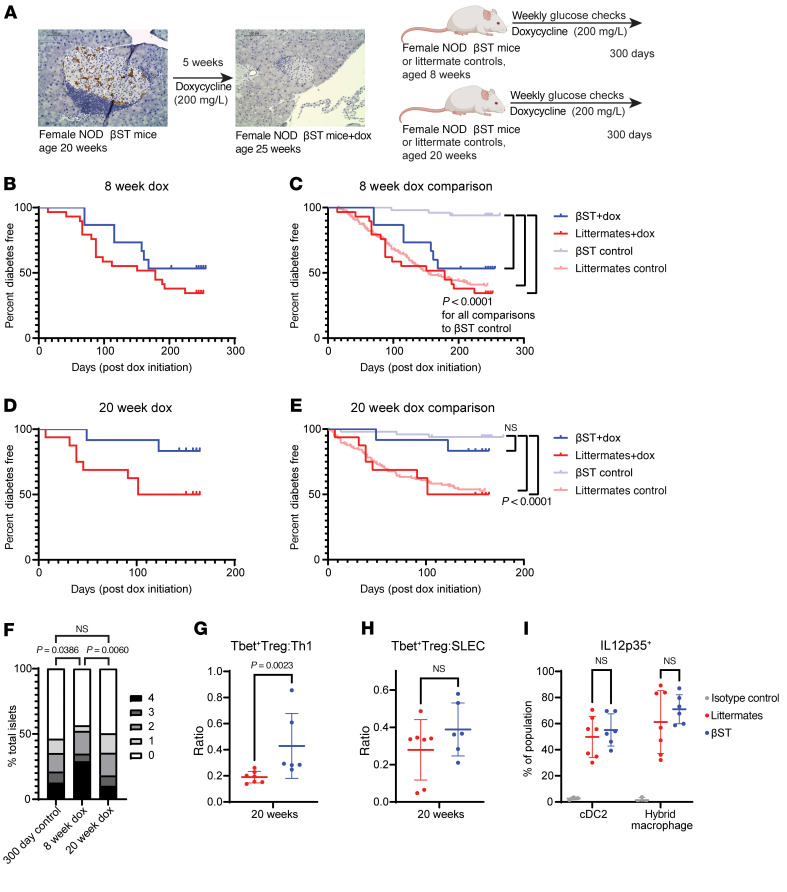
Tolerance from disease is established after 20 weeks. (**A**) Confirmation of ST8Sia6-myc shutoff after 5 weeks of doxycycline treatment by IHC. Schematic of doxycycline treatment for temporal control of ST8Sia6 expression. Scale bars: 100 μm. (**B**) Diabetes-free incidence after treatment of 15 euglycemic NOD βST and 29 euglycemic littermate mice with doxycycline starting at 8 weeks of age. (**C**) Comparison with disease kinetics of 8-week-old euglycemic non-doxycycline-treated NOD βST (*n* = 50) or littermate (*n* = 151) mice (subset from Figure 1C). (**D**) Diabetes-free incidence after treatment of 12 euglycemic NOD βST and 16 euglycemic littermate mice with doxycycline starting at 20 weeks of age. (**E**) Comparison with disease kinetics of 20-week-old euglycemic non-doxycycline-treated NOD βST (*n* = 50) or littermate (*n* = 115) mice (subset from Figure 1C). Log-rank Mantel-Cox test was used to analyze statistical differences in diabetes-free incidence of indicated groups. (**F**) Insulitis distribution in pancreas sections of NOD βST mice treated with doxycycline for 5 weeks from 8 weeks or 20 weeks of age, compared with 300-day-old pancreata from NOD βST mice never treated with doxycycline (subset from Figure 1E). Scoring and analysis as in Figure 1E. *n* = 69 islets from 12 mice (8-week doxycycline) or 127 islets from 11 mice (20-week doxycycline). (**G** and **H**) Ratio of T-bet^+^ Tregs (T-bet^+^FoxP3^+^CD4^+^) to Th1 T cells (T-bet^+^FoxP3^–^CD4^+^) (**G**) or to SLECs (T-bet^+^CD8^+^) (**H**) from islets of 20-week-old mice treated with doxycycline for greater than 5 weeks. (**I**) Quantification of IL-12p35 expression in hybrid macrophages (CD11b^+^CD11c^+^F4/80^+^Gr1^–^) and cDC2s (CD11b^+^CD11c^+^F4/80^–^Gr1^–^) from islets of 20-week-old mice treated with doxycycline for greater than 5 weeks. Same gating schemes as in Figures 4 and 5. Error bars represent SD. Mann-Whitney *U* test was used for statistical analysis between groups.

NOD βST mice possessed autoreactive T and B cells yet demonstrated insulitis that did not progress with age, suggesting that these mice developed tolerance toward β cells. Once tolerance has been established, sustained expression of ST8Sia6 to suppress an immune response may no longer be required. Our mouse was designed for doxycycline-regulatable expression of ST8Sia6 in β cells (Figure 1A). The tet-transactivator is constitutively active in the absence of doxycycline when induced by Cre as previously described; doxycycline, however, inhibits binding of tTA to tet-responsive elements in DNA. First, we verified that treatment of NOD βST mice with doxycycline in the drinking water led to the loss of the myc-tagged ST8Sia6 transgene expression in β cells (Figure 7A). To further investigate the timing for generation of local tolerance, doxycycline treatment was initiated in a cohort of euglycemic NOD βST and littermate controls at either 8 weeks (Figure 7, B and C) or 20 weeks (Figure 7, D and E) of age and continued through 300 days. Importantly, doxycycline treatment alone did not alter disease kinetics in this cohort as previous studies have suggested (53, 54), since outcomes of euglycemic littermates at both 8 and 20 weeks of age treated or never treated with doxycycline were similar. Treating euglycemic NOD βST mice with doxycycline starting at 8 weeks of age resulted in loss of protection from T1D, with a similar incidence of disease to their littermate counterparts also treated with doxycycline (Figure 7C). In contrast, euglycemic NOD βST mice treated from 20 weeks of age onward remained protected from diabetes, with only 16% disease incidence in the doxycycline-treated NOD βST group compared with 50% in the doxycycline-treated littermate group (Figure 7E). These findings were correlated with changes to insulitis severity — only euglycemic NOD βST mice treated with doxycycline starting at 8 weeks exhibited increased insulitis at 300 days, compared with NOD βST mice never treated with doxycycline or NOD βST mice treated with doxycycline starting at 20 weeks (Figure 7F).

Changes to immune infiltration in the islets of 20-week-old NOD βST mice and littermate controls treated with doxycycline for 5–8 weeks were further investigated. Under these conditions, NOD βST mice maintained a high ratio of T-bet^+^FoxP3^+^ Tregs to T-bet^+^ Th1 CD4^+^ T cells (Figure 7G), although the ratio of T-bet^+^FoxP3^+^ Tregs to CD8^+^ SLECs was normalized in comparison with littermate controls (Figure 7H). Interestingly, the previously noted reduction in IL-12p35 expression in islet-infiltrating cDC2s was lost in NOD βST mice treated with doxycycline from 20 weeks of age (Figure 7I). Taken together with earlier data, these findings establish a time frame between 8 and 20 weeks of age when β cell–specific expression of ST8Sia6 results in robust tolerance such that sialyltransferase expression may no longer be required for continued protection from T1D.

## Discussion

In this study, we demonstrated that transgenic expression of the sialyltransferase ST8Sia6 in the β cells of the islets of Langerhans protected NOD mice from autoimmune diabetes. This protection was due to the generation of peripheral tolerance that resulted in the preservation of β cell mass, with a localized reduction of the inflammatory type 1 immune response in the islets normally characteristic of T1D. We found an increase in the ratio of suppressive T-bet^+^ Tregs to effector immune cells (CD4^+^ Th1 or CD8^+^ SLECs) normally responsible for β cell rejection in NOD βST mice. Importantly, NOD βST mice and littermate controls demonstrated similar and persistent peripheral markers and mechanisms of autoimmunity against β cells. Similar levels of anti-insulin antibodies and comparably rapid induction of hyperglycemia upon anti–PD-L1 or anti–PD-1 treatment indicated that both NOD βST and littermate mice contained β cell–reactive B and T cells. However, despite the presence of autoreactive B and T cells, islets from NOD βST mice showed substantial reductions in insulitis and were protected from autoimmune destruction as compared with their littermate controls. Treatment of NOD βST mice with doxycycline to block transcriptional activation of the tet-transactivator at 20 weeks of age resulted in maintained protection from disease despite loss of ST8Sia6 expression in the β cells. These mice had comparable insulitis to NOD βST mice never treated with doxycycline and showed an elevated T-bet^+^ Treg to CD4^+^ Th1 ratio, indicating the establishment of robust tolerance. These findings support the translational potential of sialic acid–mediated immunomodulation for regulating adaptive immune cell–generated spontaneous autoimmune disease.

We previously demonstrated that ST8Sia6 generates ligands for Siglec-E in vivo and further showed expression of the human orthologs of Siglec-E, Siglec-7 and -9, on myeloid cells in the human islet (22). This previous work hypothesized that islet-resident macrophages were responsible for dampening an inflammatory response in the islets secondary to streptozotocin treatment. All myeloid populations in the islets of NOD βST mice were reduced in comparison with NOD littermates, and cDC2s and hybrid macrophages expressed the inhibitory receptor Siglec-E capable of engaging the α2,8 disialic ligands generated by ST8Sia6. Notably, these cDC2s in the islets of NOD βST mice downregulated the potent type 1 cytokine IL-12p35 in comparison with littermate controls. Previous work demonstrated that CD11c^+^ myeloid cells mediated lymphocyte trafficking to the NOD islets, with a depletion of CD11c^+^ cells strongly protecting from T and B cell infiltration into the islet (55). CD11c^+^ populations in the islets include cDC2s, hybrid macrophages, and cDC1s, all three of which were found at significantly lower numbers in the islets of NOD βST mice compared with NOD littermates. Considered alongside the reductions in the adaptive compartment of immune infiltration into the NOD βST islets, our results support β cell protection from autoimmunity both by decreased trafficking of immune cells to the islet and by reduced IL-12p35 by cDC2s that would otherwise sustain the effector function of T cells. Apart from reduced cDC2s in the draining PLNs, no other changes in the frequency or number of immune cells in the PLNs or spleens of NOD βST and littermate controls were observed. In addition, there were no changes in the PLNs and spleens in IL-12p35 expression by myeloid cells or T cell polarization toward a type 1 response in NOD βST mice compared with littermate controls.

Numerous distinct approaches are being investigated to prevent or better treat T1D, but a permanent cure remains elusive. Various attempts to modulate the immune response in T1D in humans and murine models include ongoing studies with antigen-specific CAR-Tregs (56), modulation (31, 32) or depletion (57) of T effectors, inhibition of T cell activation (58), immune checkpoint agonists (59), and B cell antagonists (60). Many of these approaches show promise, but struggle to overcome low efficacy in disease suppression, adverse secondary complications, or difficulties with scalability, among other limitations. Most studies investigating immune regulation for autoimmune suppression have focused on the adaptive arm of the immune system, predominantly the T cell effectors that eliminate their targets. However, the importance of the innate mediators of inflammation that prime the adaptive arm can be, and often is, overlooked. In contrast, the transplantation of islets into people with diabetes has shown the most promise for maintaining blood glucose levels within a physiological range. Still, this approach requires constant immunosuppression to quell both the primed immune response against β cell antigens and allograft rejection and suffers from poor long-term engraftment (61).

The presence of autoantigen-specific T cells in the blood (62) and pancreas (63) of patients with T1D has been well established. However, a recent study demonstrated that islet autoantigen-specific CD8^+^ T cells can be found in the pancreata of healthy individuals as well (64), suggesting that tolerance is broken in an islet-localized, peripheral manner during T1D pathogenesis. Our findings of islet-specific protection in NOD βST mice, supported by the ongoing progression of Sjögren’s syndrome and similar serum levels of anti-insulin autoantibodies, alongside preservation of β cell mass within the context of peripheral anti-insulin antibodies and autoreactive T cells, support the notion of Siglec–sialic acid interactions as an important checkpoint of peripheral tolerance. When utilized within the islet, immune regulation via this checkpoint may promote sustained and local tolerance in islet transplantation in people with T1D.

One risk of any immunomodulatory approach is the potential for pan-suppression of the systemic immune response. In our study, we found no evidence of off-target immune suppression, as indicated by the comparable presence of autoantibodies and ongoing Sjögren’s pathology in the submaxillary salivary glands. Future work, however, would be required to fully assess the safety of this system prior to translation to human studies. Another limitation to this work includes the imperfect representation of disease in the NOD model compared with patients. This mouse model has a well-documented female sex bias toward disease not seen in humans (36, 37), institutional heterogeneity in both timing of onset and penetrance of disease (65, 66), and imperfect translation of therapeutic approaches (26). Nonetheless, the NOD mouse remains a staple in the investigation of autoimmune disorders and T1D.

Future studies will focus on the ability of β cell expression of ST8Sia6 to modulate an already established autoimmune response. Curative islet transplantation is conducted on individuals with T1D after the disease process has already begun, as opposed to before overt disease as is the case in our transgenic mouse model. Murine transplantation studies will begin to address this by investigating the ability of islets from syngeneic or MHC-mismatched mice to prevent disease after transplantation into prediabetic NOD mice as well as to suppress or reverse active disease after transplantation into diabetic NOD mice.

In conclusion, ST8Sia6 expression in β cells of the endocrine pancreas strongly suppressed the autoimmune process behind T1D in the NOD mouse. This protection was long-lasting and generated islet-localized tolerance that persisted beyond 20 weeks of age without further expression of ST8Sia6. Alterations to both the adaptive arm of the immune system, with reductions in mediators of type 1 inflammation, and the innate arm, with reduced IL-12p35 production by cDC2s, were found in the islet but not the spleen or draining lymph nodes. These results implicate Siglec–sialic acid signaling as a worthwhile candidate in the ongoing search for translational immune regulation, whether to treat autoimmune diabetes or other inflammatory conditions.

## Methods

### Sex as a biological variable.

The NOD mouse inherently demonstrates a female sex bias in disease penetrance (36, 37). With the exception of Supplemental Figure 1, the data presented are solely from female NOD βST and littermate mice.

### Mice.

Ins2-cre (stock 003573; ref. 67) and LNL-tTA mice (stock 008600; ref. 68) were obtained from The Jackson Laboratory. Mice with myc-tagged ST8Sia6 under the control of a tetracycline-responsive element were described previously (22). These mice on the B6 background were backcrossed to NOD/ShiLtJ (stock 001976) from The Jackson Laboratory for more than 10 generations, resulting in the NOD βST and NOD littermate mice used for these studies. Mice on the NOD background were monitored via biweekly nonfasting tail vein blood glucose readings (during the light cycle) to assess for onset of disease starting at 4 weeks of age (Bayer 7377, Bayer 7311). Diabetes was defined as 3 consecutive readings greater than or equal to 250 mg/dL. The Institutional Animal Care and Use Committee at Mayo Clinic approved all animal studies performed in this work (A00003755-18-R24).

### Immune checkpoint inhibition.

Euglycemic mice were intraperitoneally injected with 500 μg anti–PD-L1 (Bio X Cell BE0101) or anti–PD-1 (Bio X Cell BE0146) on day 0, with an additional 250 μg on day 2 if the mouse was not yet hyperglycemic. Mice were monitored with daily tail vein blood glucose readings starting at time of injection, using the same definition of disease as above.

### Tet off.

Euglycemic mice were initiated on doxycycline (RPI D43020) at 200 mg/L dissolved in autoclaved deionized water, supplemented with 5 g/L of sucralose for taste. Medicated water was changed 3 times per week and provided ad libitum from study onset.

### Islet isolation.

Pancreata were isolated by perfusion of 3 mL of cold 0.9 mg/mL Collagenase P (MilliporeSigma 11249002001) dissolved in HBSS (Corning) through the common bile duct, placement of the inflated pancreas in 2 additional mL of cold 0.9 mg/mL Collagenase P, and digestion for 13 minutes at 37°C with physical agitation for 5 minutes. Islets were separated by centrifugation using a 1.077 g/mL Histopaque gradient (MilliporeSigma 10771) under RPMI 1640 (Corning), then manually picked under a dissection microscope. For flow cytometry, islets were dissociated into single cells in enzyme-free Cell Dissociation Buffer (Thermo Fisher 13151014) for 30 minutes at 37°C.

### Histology/IHC/immunofluorescence.

FFPE pancreata and submaxillary salivary glands were cut to 5 μm sections and stained via H&E or IHC. To stain for the myc tag, tissue sections underwent antigen exposure and were blocked and then stained with anti-myc (Cell Signaling CD2278S) at 1:200 overnight at 4°C, followed by HRP-conjugated secondary antibody (Biocare rmr622l) at room temperature for 40 minutes and DAB substrate for 5 minutes. Insulitis scoring was conducted on either H&E- or IHC-stained tissue sections by blinded reviewers. Islets were imaged, deidentified, and quantified on a 4-point scale (0 referring to lack of any insulitis per islet, 4 referring to circumferential insulitis around the islet). For immunofluorescence, pancreas tissue sections underwent antigen exposure and were blocked and then stained with rabbit anti-insulin (Cell Signaling C27C9) at 1:100, mouse anti-glucagon (Sigma-Aldrich G2654) at 1:500, mouse anti-myc tag (Cell Signaling 9B11) at 1:200, or rabbit anti-CD8α conjugated with AF647 (Abcam AB237365) at 1:500 at 4°C overnight; staining for insulin, glucagon, or myc was followed by secondary donkey anti-mouse Cy3 (Jackson ImmunoResearch 715-166-150) or donkey anti-rabbit FITC (Jackson ImmunoResearch 711-096-153) at 1:100 for 2 hours at room temperature. Tissues were mounted using Antifade Mounting Medium with DAPI (Vectashield H-1200-10). Whole tissues were imaged at ×5, and insulin and glucagon areas were quantified using Zen Blue (v3.5); specific islets were imaged at ×20. Sjögren’s syndrome analysis was conducted on H&E-stained sections of whole salivary gland. Number of immune foci was quantified by visualization, and percentage of gland occupied by immune infiltration was determined by ImageJ (NIH) analysis.

### ELISA.

Whole blood was collected from mice retro-orbitally using capillary tubes. The blood was left to coagulate at room temperature for 30 minutes, then centrifuged at 1,500*g* for 10 minutes at 4°C. Serum was isolated and frozen at –80°C until analysis for autoantibodies. For quantification of serum anti-insulin antibodies, 96-well plates (Thermo Fisher Scientific 3855) were coated with 100 μL of 1.0 μg/mL insulin (Sigma-Aldrich 91077C) for more than 18 hours at 4°C. Plates were washed 4 times with PBS plus 0.005% Tween-20 before use. Wells were blocked with 1% BSA (Roche 03116964001) in PBS for 1 hour at room temperature. Immediately after, 50 μL of serum was placed in each well and incubated for 1 hour at room temperature on the shaker. Wells were washed 10 times with PBS plus 0.005% Tween-20, then incubated with HRP-conjugated goat anti-mouse IgG (Southern Biotech 5300-05) at 1:1,000 in PBS plus 0.05% BSA for 1 hour at room temperature on a shaker. Wells were washed 10 times with PBS plus 0.005% Tween-20. One hundred microliters of substrate (BioLegend 421101) was added to each well and incubated at room temperature for 10 minutes; then the reaction was halted with 50 μL of TMB stop solution (Southern Biotech 0413-01). Colorimetric readout at 450 nm (Biotek) was conducted immediately after. A standard curve was generated using a commercially available mouse anti-insulin antibody (Invitrogen M1-83258). Quantification of serum insulin and C-peptide was done with precoated ELISA plates (Alpco 80-INSMS-E01, Alpco 80-CPTMS-E01) following manufacturer instructions.

### IL-12 challenge.

Recombinant IL-12 (BioLegend 577004) was injected intraperitoneally in euglycemic 14-week-old mice at 20 μg/kg daily for 10 days, or until hyperglycemic. Mice were monitored with daily tail vein blood glucose readings starting at time of injection, using the same definition of disease as above.

### Flow cytometry.

Spleen and pancreatic lymph nodes were harvested, manually dissociated, and filtered through 70 μm mesh. Splenocytes were additionally processed with ACK lysis buffer at room temperature for 3 minutes to remove red blood cells. Single cells from tissues (spleen, pancreatic lymph node, islets) were washed with PBS, blocked in 5% serum (1:1 rat and mouse serum, Invitrogen 10410 and 10710C) on ice for 10 minutes, and incubated with surface antibodies on ice for 30 minutes. In staining for transcription factors, cells were further fixed, permeabilized, then intranuclearly stained (Cytek TNB-1022, TNB-1213). In staining for cytokines, dissociated single cells were first incubated with protein transport inhibitor (Invitrogen 00-4980-93) diluted to 1× in RPMI at 37°C for 3 hours, then washed in PBS and blocked in 5% serum. Cells were fixed, permeabilized, and intracellularly stained (BD 554714). Samples were run using an Attune NxT flow cytometer (Thermo Fisher Scientific) or Cytek Aurora 5L (Cytek).

The following antibodies were used: CD45-BUV395 (BD 564279 at 1:1,000), L/D-UV450 (Tonbo 13-0868 at 1:1,000), CD11c-BUV615 (BD 751222 at 1:100), CD11b-PacBlue (BioLegend 562894 at 1:200), Ly6C-BV480 (BioLegend 569439 at 1:500), CD8b-BV785 (BioLegend 126631 at 1:1,000), Ly6G-BV785 (BioLegend 127645 at 1:200), CD4-SparkBlue (BioLegend 100494 at 1:1,000), T-bet–RB780 (BD 569089 at 1:100), F4/80-ef660 (Invitrogen 50-4801-82 at 1:200), IFN-γ–APC (BioLegend 505810 at 1:100), isotype control–APC (BD 554686 at 1:100), FoxP3-APC (Tonbo 20-5773 at 1:100), CD11c-BV421 (BioLegend 117330 at 1:500), RORγt-BV421 (BD 562894 at 1:100), MHCII-BV510 (BioLegend 107635 at 1:400), L/D-BV510 (Tonbo 13-0870 at 1:1,000), CD8a-BV510 (BioLegend 100752 at 1:500), TCRβ-BV605 (BioLegend 109241 at 1:200), CD45-BV785 (BioLegend 103149 at 1:500), CD8a-BV785 (BioLegend 100750 at 1:500), CD11b-FITC (BioLegend 101206 at 1:200), CD45-FITC (BioLegend 103108 at 1:500), L/D-GR780 (Tonbo 13-0865 at 1:1,000), IL-12p35–PE (R&D Systems IC2191P at 1:100), isotype control–PE (BioLegend 400408 at 1:100), Siglec-E–PE–Cy7 (BioLegend 677108 at 1:100), isotype control–PE–Cy7 (BioLegend 400522 at 1:100), T-bet–PE–Cy7 (BioLegend 644824 at 1:500), F4/80-PE-Dazzle (BioLegend 123146 at 1:400), Gr1-PerCP (BioLegend 108426 at 1:500), CD4-PerCP (BioLegend 100538 at 1:500), PD-1–APC–Fire750 (BioLegend 135240 at 1:200), and PD-L1–BV421 (BioLegend 124315 at 1:100).

### Statistics.

Figures were generated using Adobe Illustrator (v28) and GraphPad Prism (v10, GraphPad Software LLC). Data presented represent mean ± SD. Statistical tests were calculated in GraphPad Prism and included log-rank Mantel-Cox test, χ^2^ test, Mann-Whitney *U* test, and 1-way ANOVA. A *P* value less than 0.05 was considered significant.

### Study approval.

All animal studies were reviewed and approved by and conducted in accordance with the Institutional Animal Care and Use Committee at Mayo Clinic (A00003755-18-R24).

### Data availability.

Values for all plotted data are available in the Supporting Data Values file.

## Author contributions

JC, PB, AM, and VSS designed the study. MJS generated the mice used in these studies. JC, SC, TN, DF, AZ, and MR performed the experiments. MR, BH, and CW conducted animal husbandry. JC and VS analyzed the data. JC generated the figures. JC and VSS wrote the manuscript. All authors approved the manuscript.

## Supplementary Material

Supplemental data

Supporting data values

## Version 1 08/01/2025

Electronic publication
