# Supplementary material for: ST8Sia6 overexpression protects pancreatic β cells from spontaneous autoimmune diabetes in nonobese diabetic mice
Source: J Clin Invest. 2025 Aug 1;135(15):e181207. doi: 10.1172/JCI181207 (PMC12321382; doi:10.1172/JCI181207)
Supplement: Supplemental data [file jci-135-181207-s233.pdf]

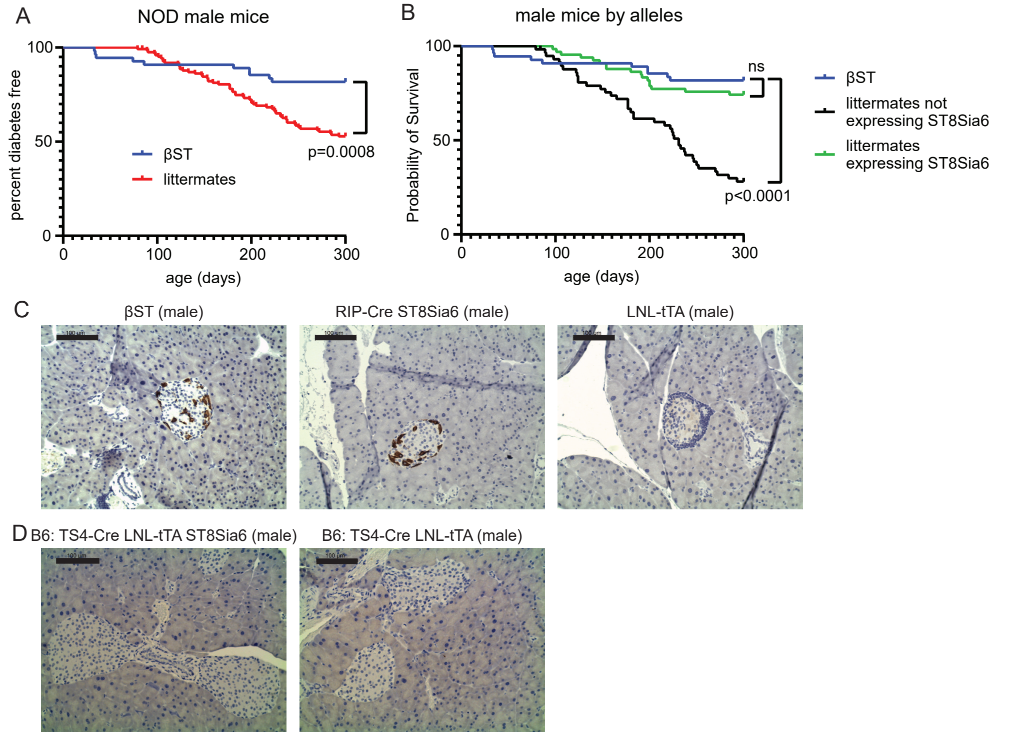

Supplemental Figure 1: Male littermate mice on the NOD background demonstrate leaky expression of the ST8Sia6 transgene. (A) Kaplan Meier curve for diabetes free incidence in male NOD βST and NOD littermate mice. n = 55 (βST) or 124 (littermates). Statistical significance was determined by Log-rank Mantel Cox test. (B) Sub-analysis by allelic expression of the littermate population from (A) for diabetes free incidence. n = 55 (βST), 58 (littermates without ST8Sia6), or 66 (littermates with ST8Sia6). Log-rank Mantel Cox test was used for statistical analysis in comparisons against βST. (C) Representative IHC imaging for the myc tag in pancreatic sections from male NOD mice. Both male βST NOD mice and RIP-Cre ST8Sia6 allele (without LNL-tTA) positive male littermate NOD mice stain positive in the islets. Scale bar = 100μm. (D) Representative IHC imaging for the myc tag in pancreatic sections from male B6 mice containing (left) or lacking (right) the ST8Sia6 allele. The LNL-tTA and ST8Sia6 alleles are identical to the βST mice, though this model is driven by TS4-Cre instead of RIP-Cre. Scale bar = 100μm.

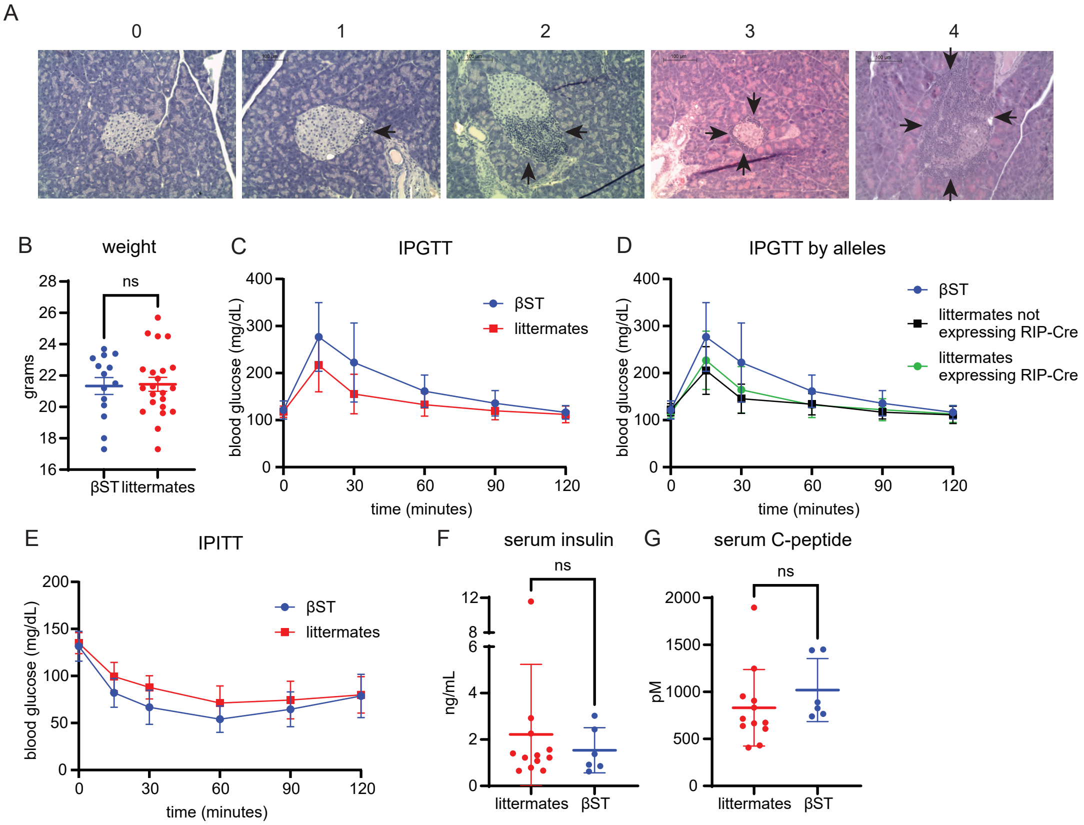

Supplemental Figure 2: βST mice are morphologically and physiologically similar to littermates. (A) Representative H&E staining of islet scores used for calculation of insulitis in Figures 1, 3, and 7. Arrows highlight peri-islet insulitis, noted by hematoxylin dense regions of immune cell infiltration. (B) Weight distribution of euglycemic NOD βST and NOD littermate mice between 8-10 weeks of age. (C, D) Blood glucose response after IP glucose tolerance test (IPGTT) in euglycemic 8-10 week old NOD βST and NOD littermate mice (D), with littermate mice differentiated by expression of the RIP-Cre element (E). n = 14 (NOD βST) or 21 (littermates, 10 not expressing RIP-Cre, 11 expressing RIP-Cre). (E) Blood glucose response after IP insulin tolerance test (IPITT) in euglycemic 8-10 week old NOD βST and NOD littermate mice. n = 10 (NOD βST) or 6 (littermates). (F, G) Serum levels of insulin and C-peptide in euglycemic 8-10 week old NOD βST and NOD littermate mice. n = 6 (NOD βST) or 12 (littermates). Mann-Whitney U test was used for comparisons.

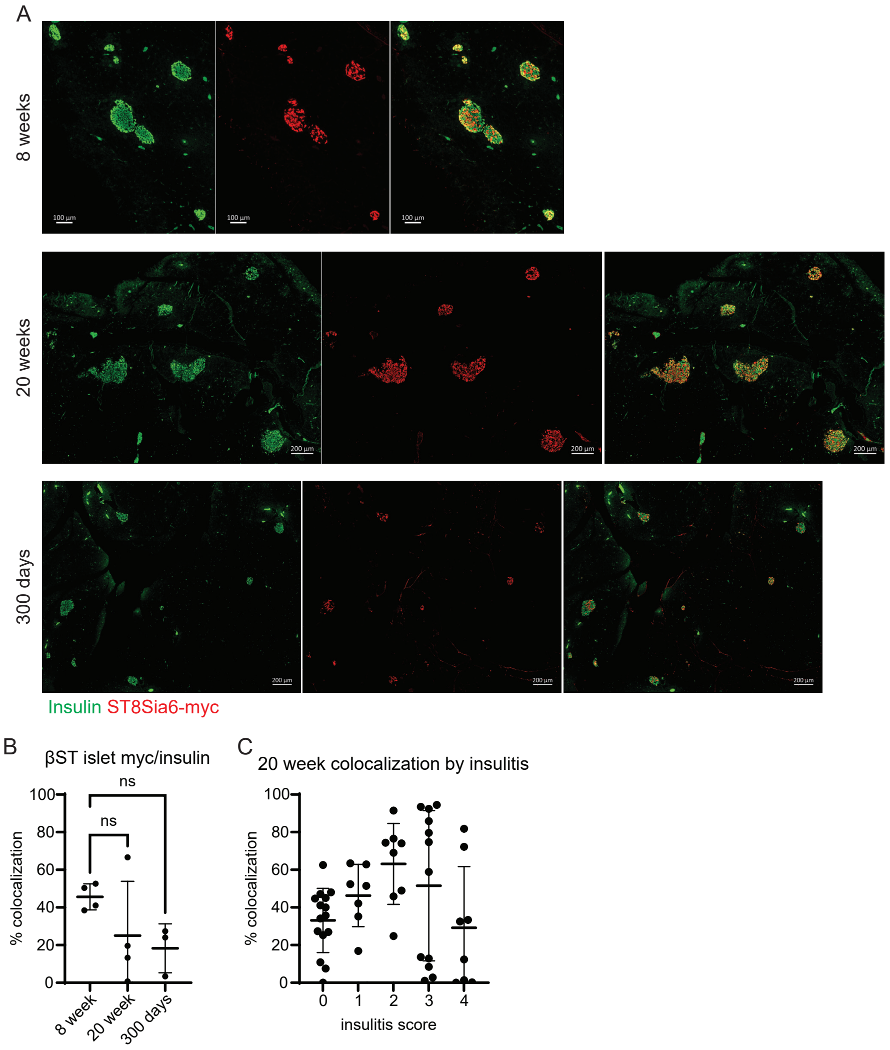

Supplemental Figure 3: Islets from NOD βST mice have similar expression of ST8Sia6 that does not decrease with insulitis. (A) Immunofluorescence images of histologic sections of pancreas from euglycemic NOD βST littermate mice at the indicated ages with insulin in green (FITC) and ST8Sia6-myc tag in red (Cy3). 3-4 pancreata from each group at each indicated age were stained and imaged, and representative islets are presented with quantification of colocalization in (B). Scale bar as indicated. n = 4 (8 weeks), 4 (20 weeks), or 3 (300 days). One way ANOVA was performed for statistical analysis between all 3 groups. (C) Correlation of insulitis score with coexpression of ST8Sia6-myc with insulin from 20 week old mice. n = 15 islets (score 0), 7 (score 1), 8 (score 2), 12 (score 3), or 8 (score 4).

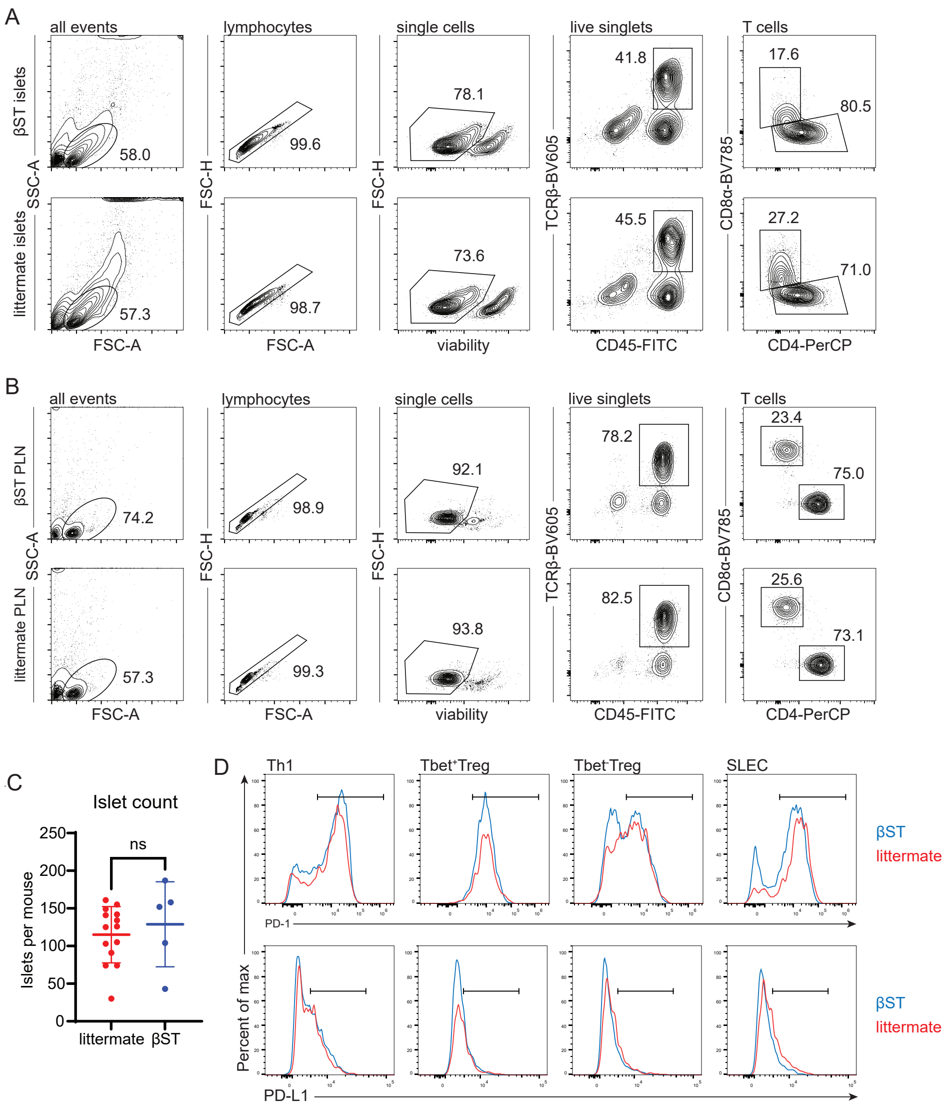

Supplemental Figure 4: Representative gating trees for identification of T cell populations and assessment of the type 1 immune response in the islets (A) and PLNs (B) of euglycemic 14 week old NOD βST and NOD littermate mice. (C) Number of islets harvested per NOD βST and NOD littermate mice for flow cytometric analysis in Figure 4. (D) Representative histograms of PD-1 and PD-L1 expression in T cells subsets analyzed in Figure 4.

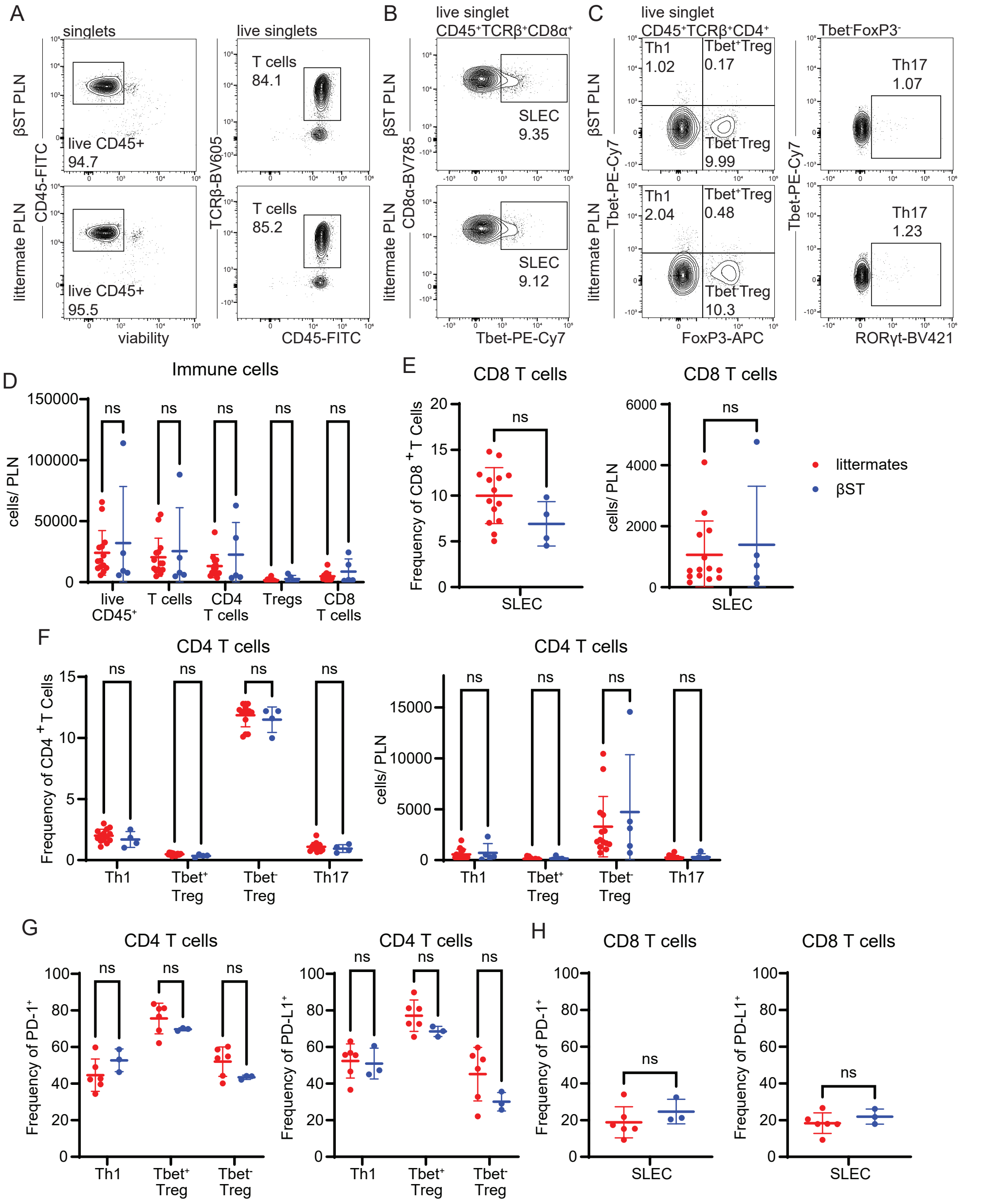

Supplemental Figure 5: NOD βST mice have no differences in immune mediators in the draining pancreatic lymph node. Representative flow cytometry plots of immune cells (A), short lived effector CD8 T cells (SLECs) (B), and CD4 T cell subsets (C) in pancreatic lymph nodes from euglycemic 14 week old NOD βST (top) or NOD littermate (bottom) mice. Cells were previously gated on size and singlets. (D) Quantification of normalized event count of cells per PLN depicted in (A). The number of each cell type was normalized to the total number of pancreatic lymph nodes (1 or 2) isolated per mouse. (E) Quantification of frequency and normalized cell count of SLECs per PLN from (B) per mouse. (F) Quantification of frequency and normalized cell count of CD4 T cell subsets per PLN from (C) per mouse. n = 5 (NOD βST) or 14 (littermates). Populations were defined as in Figure 5. (G, H) Quantification of PD-1 and PD-L1 expression in CD4 T cell subsets (G) and SLECs (H) in PLNs from euglycemic 14 week old NOD βST or NOD littermate mice. n = 3 (NOD βST) or 6 (littermates). Error bars represent standard deviation from the mean. Mann-Whitney U tests were performed for statistical significance between populations.

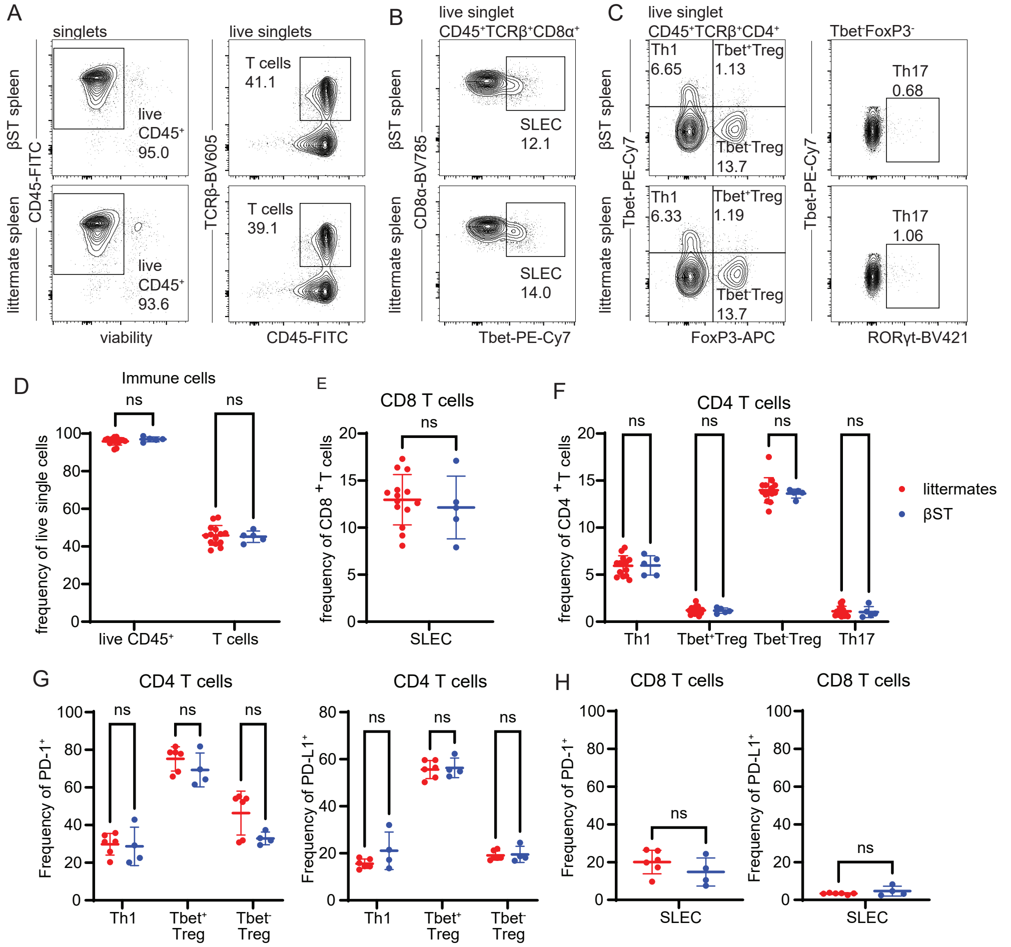

Supplemental Figure 6: βST mice have no differences in type 1 immune mediators in the spleen. Representative flow cytometry plots of immune cells (A), short lived effector CD8 T cells (SLECs) (B), and CD4 T cell subsets (C) in the spleens from euglycemic 14 week old NOD βST (top) or NOD littermate (bottom) mice. Cells were previously gated on size and singlets. (D) Frequency of cells depicted in (A) in the spleen. 10^6 processed cells were stained. (E) Quantification of frequency of SLECs from spleens shown in (B). (F) Quantification of frequency of CD4 T cell subsets from spleens shown in (C). n = 5 (βST) or 14 (littermates). Populations were defined as in Figure 5. (G, H) Quantification of PD-1 and PD-L1 expression in CD4 T cell subsets (G) and SLECs (H) in spleens from euglycemic 14 week old NOD βST or NOD littermate mice. n = 4 (NOD βST) or 6 (littermates). Error bars represent standard deviation from the mean. Mann-Whitney U tests were performed for statistical significance between populations.

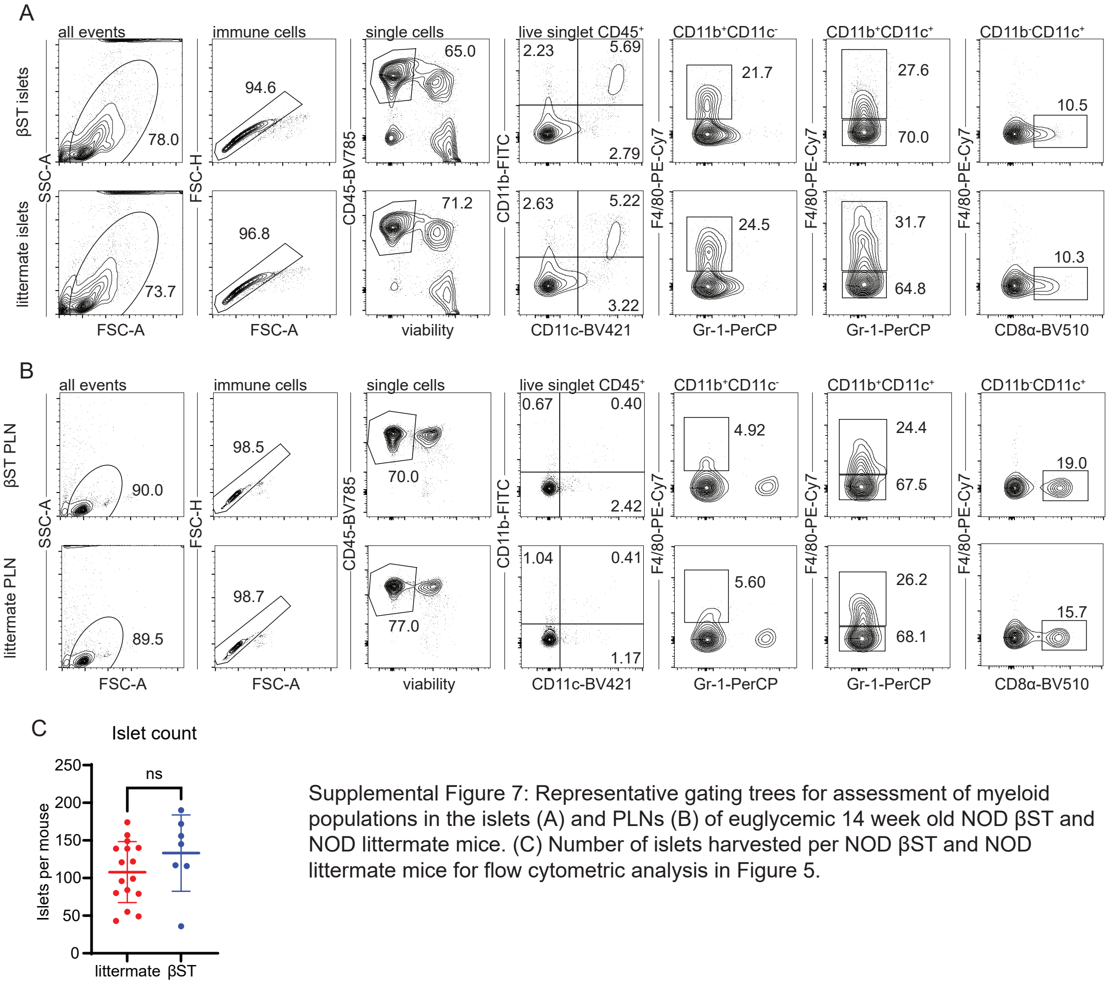

Supplemental Figure 7: Representative gating trees for assessment of myeloid populations in the islets (A) and PLNs (B) of euglycemic 14 week old NOD βST and NOD littermate mice. (C) Number of islets harvested per NOD βST and NOD littermate mice for flow cytometric analysis in Figure 5.

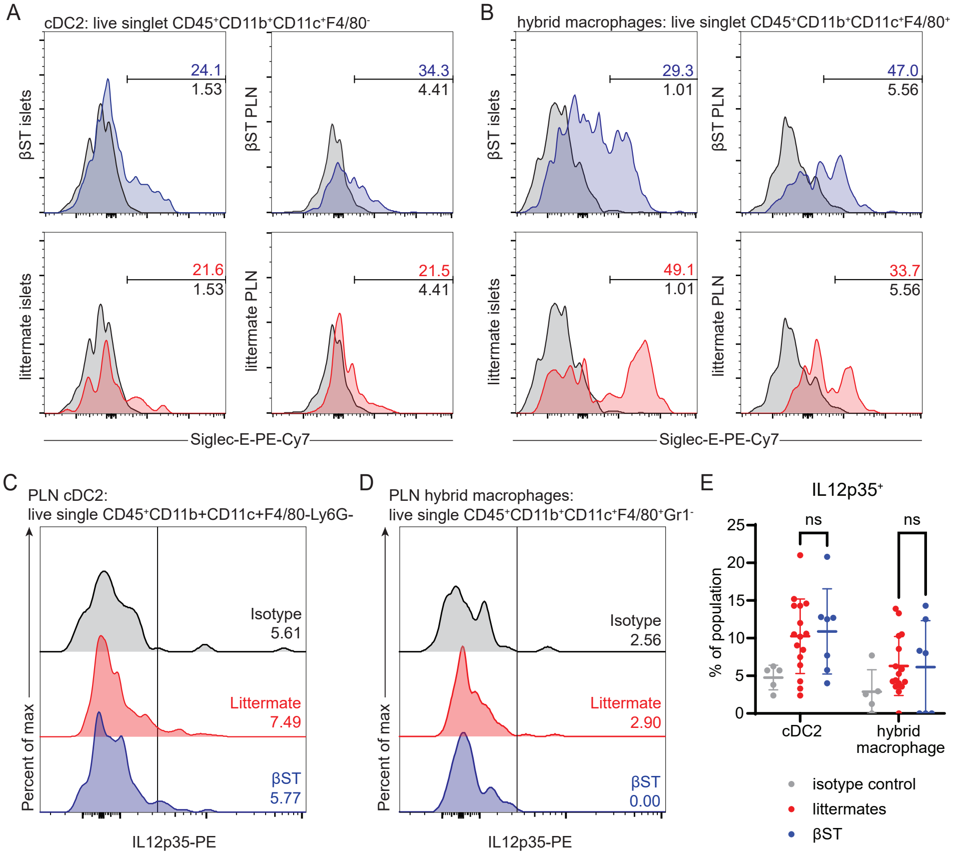

Supplemental Figure 8: Some myeloid cells within the islets express Siglec-E. (A) Representative flow cytometry histograms of Siglec-E expression in cDC2s from islets (left) or PLNs (right) of euglycemic 14 weeks old NOD βST and NOD littermate mice, compared to isotype control (grey). (B) Representative flow cytometry histograms of Siglec-E expression in hybrid macrophages from islets (left) or PLNs (right) of euglycemic 14 weeks old NOD βST and NOD littermate mice, compared to isotype control (grey). Representative flow cytometry histogram of IL12p35 expression in cDC2s (C) or hybrid macrophages (D) from the PLNs of euglycemic 14 weeks old NOD βST and NOD littermate mice, compared to isotype control (grey). Vertical line denotes the positive gate. (E) Quantification of data shown in (C) and (D). n = 7 (βST), 16 (littermates), or 5 (isotype control). Error bars represent standard deviation from the mean. Mann-Whitney U tests were performed for statistical significance between populations.
